# Atlas-guided discovery of transcription factors for T cell programming

**DOI:** 10.1038/s41586-025-09989-7

**Published:** 2026-02-04

**Authors:** H. Kay Chung, Cong Liu, Anamika Battu, Alexander N. Jambor, Brandon M. Pratt, Fucong Xie, Brian P. Riesenberg, Eduardo Casillas, Ming Sun, Elisa Landoni, Yanpei Li, Qidang Ye, Daniel Joo, Jarred Green, Zaid Syed, Nolan J. Brown, Matthew Smith, Shixin Ma, Shirong Tan, Brent Chick, Victoria Tripple, Z. Audrey Wang, Jun Wang, Bryan Mcdonald, Peixiang He, Qiyuan Yang, Timothy Chen, Siva Karthik Varanasi, Michael A. LaPorta, Thomas H. Mann, Dan Chen, Filipe Hoffmann, Josephine Ho, Jennifer Modliszewski, April Williams, Yusha Liu, Zhen Wang, Jieyuan Liu, Yiming Gao, Zhiting Hu, Ukrae H. Cho, Longwei Liu, Yingxiao Wang, Diana C. Hargreaves, Gianpietro Dotti, Barbara Savoldo, Jessica E. Thaxton, J. Justin Milner, Susan M. Kaech, Wei Wang

**Affiliations:** 1 NOMIS Center for Immunobiology and Microbial Pathogenesis, Salk Institute for Biological Studies, La Jolla, CA, USA.; 2 Lineberger Comprehensive Cancer Center, University of North Carolina at Chapel Hill, Chapel Hill, NC, USA.; 3 Department of Cell Biology and Physiology, University of North Carolina at Chapel Hill, Chapel Hill, NC, USA.; 4 Department of Chemistry and Biochemistry, University of California San Diego, La Jolla, CA, USA.; 5 Bioinformatics and Systems Biology Program, University of California San Diego, La Jolla, CA, USA.; 6 Department of Pharmacology, University of North Carolina at Chapel Hill, Chapel Hill, NC, USA.; 7 Molecular and Cell Biology Laboratory, Salk Institute for Biological Studies, La Jolla, CA, USA.; 8 Alfred E. Mann Department of Biomedical Engineering, University of Southern California, Los Angeles, CA, USA.; 9 Department of Bioengineering, Institute of Engineering in Medicine, University of California, La Jolla, CA, USA.; 10 Razavi Newman Integrative Genomics and Bioinformatics Core, Salk Institute for Biological Studies, La Jolla, CA, USA.; 11 Halıcıoğlu Data Science Institute, University of California San Diego, La Jolla, CA, USA.; 12 Department of Electrical and Computer Engineering, Texas A&M University, College Station, TX, USA.; 13 Department of Microbiology and Immunology, University of North Carolina at Chapel Hill, Chapel Hill, NC, USA.; 14 Department of Cellular and Molecular Medicine, University of California San Diego, La Jolla, CA, USA.; 15 Present address: Institute for Immunology, Allen Institute, Seattle, WA, USA.; 16 These authors contributed equally: H. Kay Chung, Cong Liu.

## Abstract

CD8^+^ T cells differentiate into diverse states that shape immune outcomes in cancer and chronic infection^1–4^. To define systematically the transcription factors (TFs) driving these states, we built a comprehensive atlas integrating transcriptional and epigenetic data across nine CD8^+^ T cell states and inferred TF activity profiles. Our analysis catalogued TF activity fingerprints, uncovering regulatory mechanisms governing selective cell state differentiation. Leveraging this platform, we focused on two transcriptionally similar but functionally opposing states that are critical in tumour and viral contexts: terminally exhausted T (TEX_term_) cells, which are dysfunctional^5–8^, and tissue-resident memory T (T_RM_) cells, which are protective^9–13^. Global TF community analysis revealed distinct biological pathways and TF-driven networks underlying protective versus dysfunctional states. Through in vivo CRISPR screening integrated with single-cell RNA sequencing (in vivo Perturb-seq) we delineated several TFs that selectively govern TEX_term_ cell differentiation. We also identified HIC1 and GFI1 as shared regulators of TEX_term_ and T_RM_ cell differentiation and KLF6 as a unique regulator of T_RM_ cells. We discovered new TEX_term_-selective TFs, including ZSCAN20 and JDP2, with no previous known function in T cells. Targeted deletion of these TFs enhanced tumour control and synergized with immune checkpoint blockade but did not interfere with T_RM_ cell formation. Consistently, their depletion in human T cells reduces the expression of inhibitory receptors and improves effector function. By decoupling exhaustion T_EX_-selective from protective T_RM_ cell programmes, our platform enables more precise engineering of T cell states, accelerating the rational design of more effective cellular immunotherapies.

Cell states are the range of cellular phenotypes arising from a defined cell type’s interaction with its environment. Within the immune system, T cells possess several differentiation states, particularly as naive T cells differentiate into diverse states with different functionalities and trafficking patterns in various immune environments, such as tumours and virus infections^1–4^. As transcription factors (TFs) govern cell state differentiation^14^, understanding how TFs shape these states is essential for programming beneficial states with therapeutic potential. One promising application of cell state engineering is enhancing CD8^+^ T cells for adoptive cell transfer therapy (ACT) of tumour-infiltrating lymphocytes (TILs) or chimeric antigen receptor (CAR) T cells. However, identifying TFs that control CD8^+^ T cell states is difficult owing to substantial heterogeneity and overlapping transcriptomes, even between functionally divergent states.

We focused on two transcriptionally similar yet functionally divergent states: the protective functional tissue-resident memory (T_RM_) cell state and the dysfunctional terminally exhausted (TEX_term_) cell state. Many studies show that TILs with T_RM_ cell characteristics correlate with better survival in patients with solid tumours^9–13^. Conversely, during persistent antigen stimulation scenarios such as chronic virus infection (for example, HIV) or cancer, T cells progressively express diverse inhibitory receptors, including PD1, and lose memory potential and effector functions. This process is known as T cell exhaustion (TEX)^5–8^, and cells in this trajectory eventually adopt the TEX_term_ cell state. TEX_term_ cells express higher levels of diverse inhibitory receptors (for example, TIM3 and CD101), lack effector and proliferative capacity, and do not respond effectively to immune checkpoint blockade (ICB), such as anti-PD1 monoclonal antibody (mAb) blockade^15–17^. High TEX_term_ cell marker expression often indicates poor prognosis in solid tumours, although some markers also correlate with ICB response, highlighting their complex role in tumour immunity^18,19^. Despite their distinct functional effects on cancer outcomes, TEX_term_ and T_RM_ cells both reside preferentially in tissues^1,3^ and display remarkable similarities in their transcriptional profiles, including key regulatory TFs such as BLIMP1 (refs. 5,20–22), BHLHE40 (refs. 23,24) and NR4A2 (refs. 9,25,26) (Fig. 1a,b and Extended Data Fig. 1a–c). These two cell states even exhibit highly correlated open chromatin regions (Extended Data Fig. 1d), complicating the precise identification of TFs whose disruption may selectively inhibit TEX_term_ cell development while preserving T_RM_ cell development. Given that many TFs are expressed commonly across different CD8^+^ T cell states and differentiation trajectories, a sophisticated and precise bioinformatics approach is crucial to pinpoint the bona fide cell-state-specifying TFs that are essential for T cell programming.

We hypothesized that key TFs controlling selective CD8^+^ T cell differentiation could be identified through systematic comparison of TF activity across the differentiation landscape. Accurate prediction requires recognizing that TF activity does not necessarily mirror expression, as it depends on post-translational modifications, cofactors and target accessibility^27^, and that TF effects propagate through genetic networks. We therefore developed a multi-omics atlas integrating transcriptomic and chromatin accessibility data from nine CD8^+^ T cell states to understand ‘global’ influences of TFs in each cell state and to identify ‘selective’ or ‘shared’ TFs. Our atlas-based platform can map TF communities and their target genes (‘regulatees’), guiding state-specific differentiation.

## Multi-omics atlas maps of CD8^+^ T cell TFs

**Fig. 1 |:**
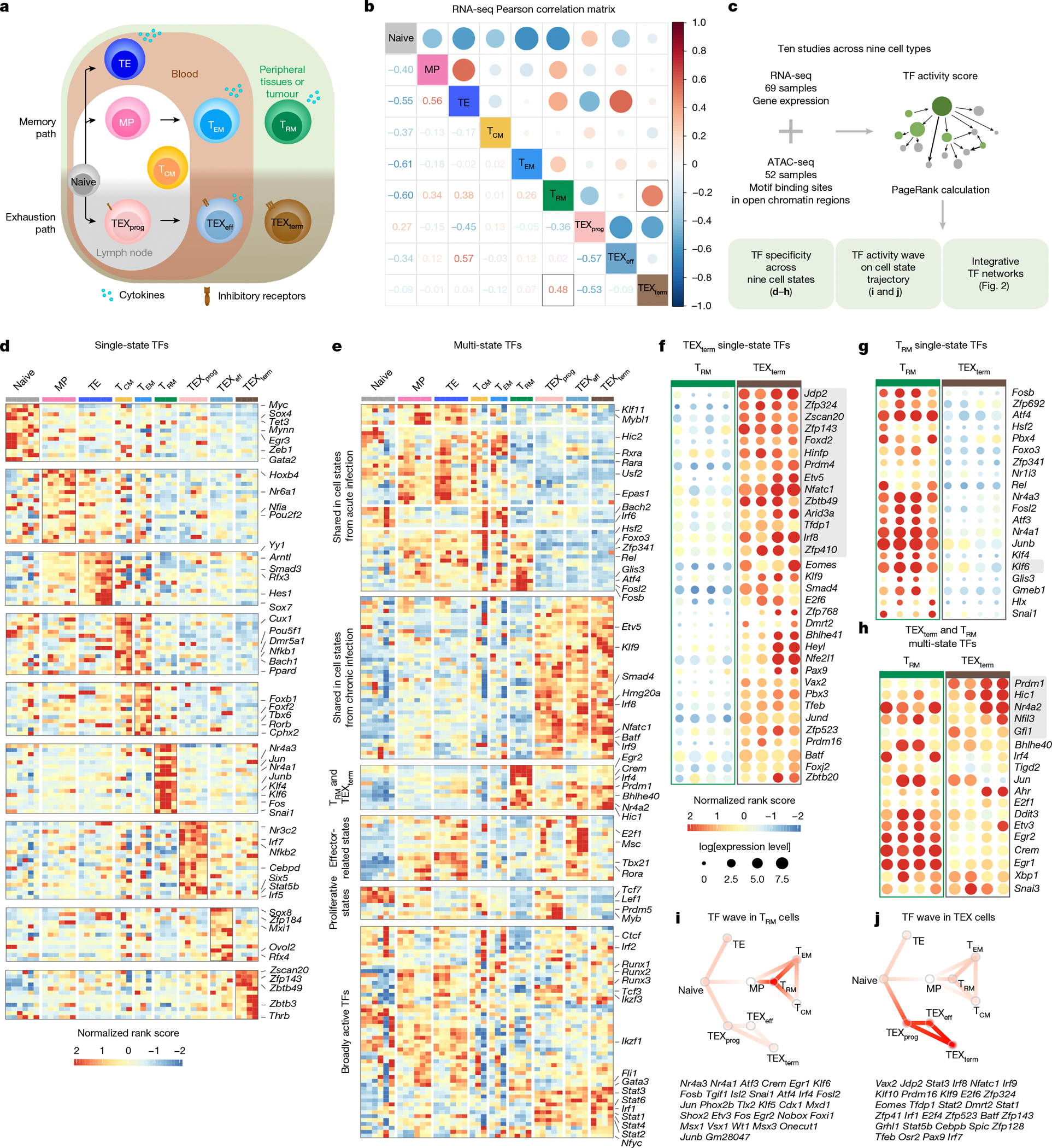
Transcriptional and epigenomic atlas of CD8^+^ T cell differentiation states and TF identification pipeline. **a**, Diagram summarizing CD8^+^ T cell trajectories during acute and chronic infection or tumour, highlighting differentiation into various effector, memory and exhaustion states, including parallel T_RM_ and TEX_term_ lineages with overlapping tissue localization. **b**, Pearson correlation matrix of batch-effect-corrected RNA-seq datasets. Both colour intensity and circle size indicate correlation strength, with red denoting the highest correlation. **c**, Workflow of the integrative Taiji analysis. Matched RNA-seq and ATAC-seq datasets^3,9,17,22,31–35^ were used to construct a regulatory network and calculate TF activity scores using PageRank. Downstream analysis included identification of single- and multi-state TFs, TF ‘waves’ and network communities. **d**–**h**, TFs (rows) and samples (columns) are displayed as *z*-normalized PageRank heatmaps. Each column corresponds to a dataset. **d**,**e**, PageRank scores of genes encoding 136 single-state TFs (**d**) and 173 multi-state TFs (**e**). **f**–**h**, Bubble plots show normalized TF PageRank scores and expression for genes encoding TEX_term_-selective (**f**), T_RM_ -selective (**g**) and multi-state (**h**) TFs that are active in both cell states. Circle colour represents the normalized PageRank score (red, high) and circle size indicates log mRNA expression across five datasets. TFs are ordered by *P* value; validated TF genes are highlighted in grey. **i**,**j**, TF ‘waves’ associated with exhaustion (**i**) or T_RM_ cell differentiation (**j**), indicating coordinated activity of TF groups during cell state transitions. Sample sizes and statistical details for cell state definitions and TF selection criteria are provided in Extended Data Figs. 1e and 2a, respectively.

Our initial objective was to create a comprehensive catalogue of TF activity across diverse CD8^+^ T cell states by integrating our TF activity analysis pipeline, Taiji^28–30^, with comparative statistical analysis. In Taiji, the gene regulatory network (GRN) is a weighted, directed network that models regulatory interactions between TFs and their target genes. In this GRN, each node corresponds to a gene, and its weight is proportional to the gene’s expression level. Each edge represents a regulatory interaction and is weighted on the basis of a combination of factors: the predicted binding affinity of the TF to the target gene, chromatin accessibility at the target gene’s locus and the expression levels of both the TF and the target gene^28,29^ (Fig. 1c). To determine the global influence of each TF within the network, Taiji applies a personalized PageRank algorithm, which assigns an ‘importance’ score to each node that is based on both the quantity and quality of incoming connections. This approach yields a measure of TF activity that reflects the influence of each TF in the broader regulatory landscape, accounting for upstream regulators, downstream targets and feedback loops through iterative computation.

With Taiji, we previously identified TFs involved in pan-immune lineage commitment, including natural killer cells, dendritic cells, B cells and γδ T cells^30^. Although earlier studies provided foundational insights into cell differentiation, a more refined analysis within CD8^+^ T cells is needed to achieve higher resolution of TF roles. Therefore, leveraging the improved statistical filtering, we aimed to quantify the global influence of TFs across all CD8^+^ T cell states.

**Extended Data Fig. 1 |:**
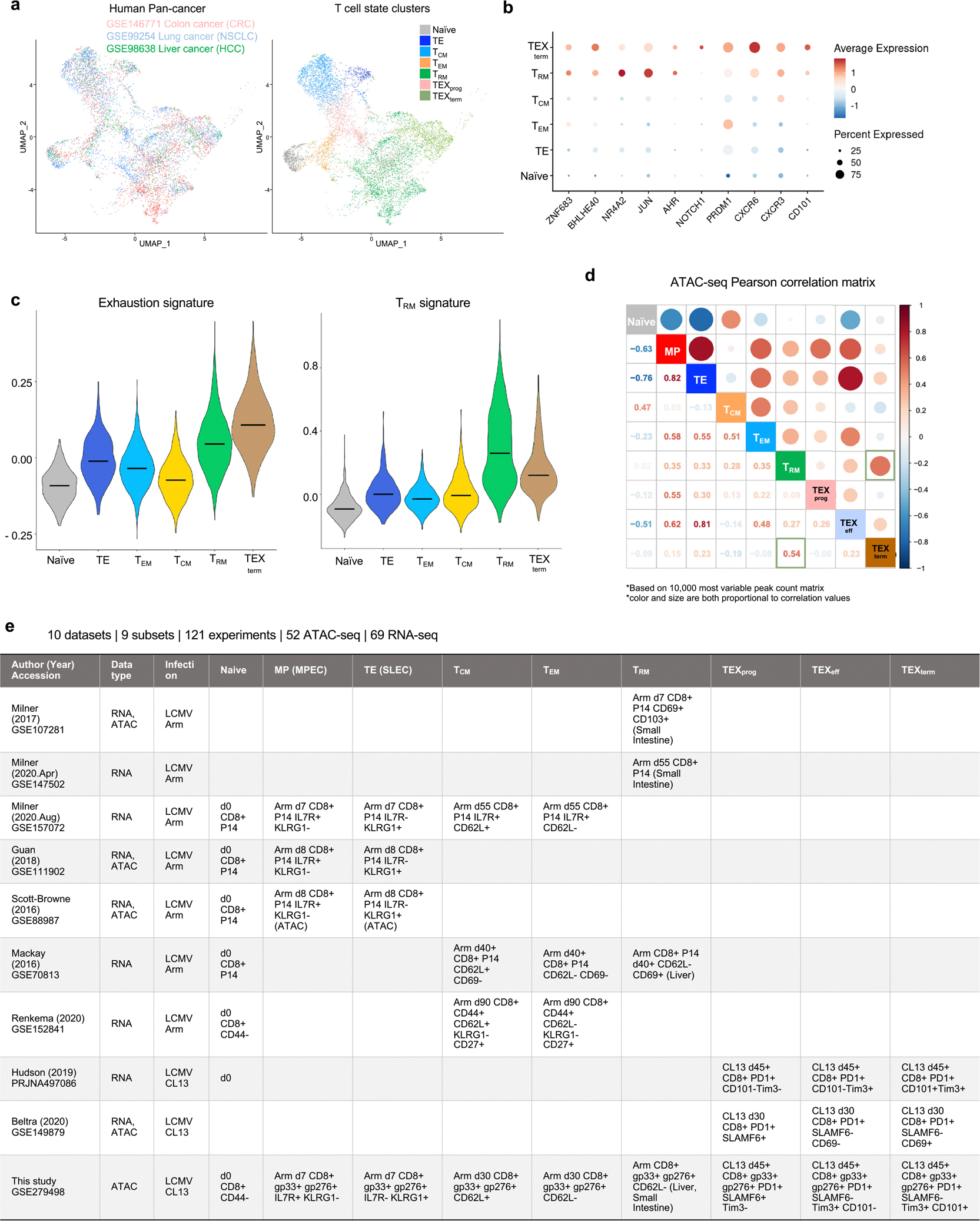
Parallel differentiation of T_RM_ and TEX_term_ and their transactional and epigenetic similarity. **a**, UMAP of scRNA-seq data of T cells from blood, tumor, and adjacent normal tissues of CRC^85^, NSCLC^44^, and HCC^86^ patients. Unbiased clustering identified multiple T cell states consistent with those observed murine LCMV infection and tumors. **b**, T_RM_ marker genes show higher expression in TEX_term_ cluster from Pan-cancer scRNA-seq in **a**. **c**, Both T_RM_ and TEX_term_ clusters upregulate exhaustion-^8^ and T_RM_^31^-associated gene signatures. **d**, Pearson correlation matrix of batch-corrected ATAC-seq datasets^3,9,33,34^. Color and size are both proportional to correlation strength. **e**, A total of 121 experiments across multiple data sets^3,9,17,22,31–35^ were utilized to generate an epigenetic and transcriptional atlas of CD8^+^ T cells under chronic and acute antigen exposure.

To begin, we analysed assay for transposase-accessible chromatin using sequencing (ATAC-seq) and RNA sequencing (RNA-seq) datasets from 121 CD8^+^ T cell samples spanning nine distinct states, using both previously published and newly generated datasets from well-characterized acute and chronic lymphocytic choriomeningitis virus (LCMV) infections^3,9,17,22,31–35^ (Extended Data Fig. 1e and Supplementary Table 1). In acute LCMV–Armstrong infections, CD8^+^ T cells differentiate into memory precursor (MP), terminal effector (TE), effector memory (T_EM_), central memory (T_CM_) and T_RM_ states. In chronic LCMV–Clone 13 infections, they adopt heterogeneous exhaustion cell states, including progenitors of exhaustion (TEX_prog_), effector-like exhaustion (TEX_eff_) and TEX_term_ states (Fig. 1a).

**Extended Data Fig. 2 |:**
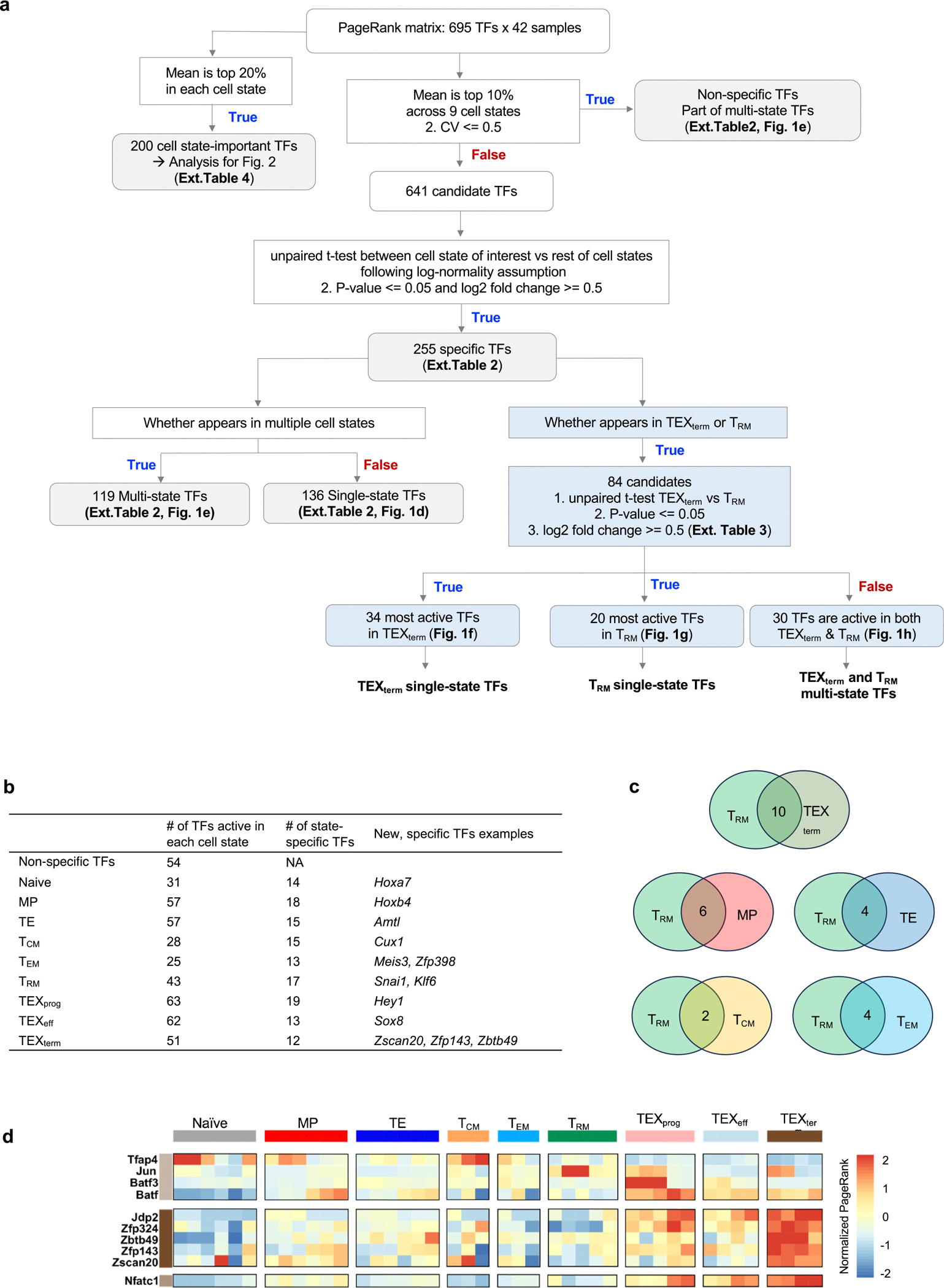
Cataloging key TFs across CD8^+^ T cell states. **a**, Logic flow of the unbiased PageRank comparison to classify single-state/multi-state TFs. **b**, Number of TFs catalogued in each cell state. **c**, Venn Diagrams showing overlap of TFs with the T_RM_ cell state. **d**, TF activity score (normalized PageRank) of previously reported TEX_term_-preventing TFs (TFAP4, JUN, BATF3, and BATF), newly identified TEX_term_ single-state TFs (JDP2, ZFP324, ZBTB49, ZFP143, ZSCAN20), and NFATC1, a known TEX_term_-associated TF.

Next, we conducted an unbiased comparative analysis using statistical filtering to understand the specificity of TF activity across the CD8^+^ T cell states (Extended Data Fig. 2a and Supplementary Table 2). This identified TF genes, of which 136 were predominantly ‘single-state’ TF genes, with each cell state selectively containing 12–19 unique TF genes (Fig. 1d and Extended Data Fig. 2b). This category included new TF genes such as *Hoxa7* in naive T cells, *Snai1* in T_RM_, *Hey1* in TEX_prog_, *Sox8* in TEX_eff_, and *Zscan20* and *Jdp2* in TEX_term_ cells. By contrast, 173 TFs, including *Tcf7* and *Tbx21*, were key regulators in more than one cell state, termed ‘multi-state’ TF genes (Fig. 1e). TCF7 is a known driver of naive, MP and TEX_prog_ states, all of which are multipotent with high proliferative capacity^3,17^. Genes encoding multi-state TFs such as *Vax2*, *Batf*, *Irf8* and *Stat1* were more enriched within the exhaustion-associated cell states (TEX_prog_, TEX_eff_ and TEX_term_). Consistent with the similarity between TEX_term_ and T_RM_ cells (Fig. 1b and Extended Data Fig. 1b–d), these two cell states share the most TF genes compared with other cell states (for example, *Egr2*, *Crem* and *Prdm1*; Extended Data Fig. 2c).

Although Taiji provides a statistically grounded approach for inferring TF activity (Extended Data Fig. 2a), there is no absolute threshold for defining cell state specificity, and some misclassification is expected, particularly for TFs with overlapping functions or modest differences in activity. Still, Taiji is useful to highlight TFs with activity patterns enriched in specific cell states. For instance, although *Eomes* is classified as a TEX_term_ single-state TF gene herein, it also functions in effector, T_EM_, T_CM_ and T_RM_ cell differentiation^36,37^. This illustrates that more accurate classifications require further investigation and resolution, as performed herein for several TFs.

## TF state selectivity in TEX_term_ and T_RM_ cells

Despite the strong transcriptional overlap between TEX_term_ and T_RM_ cells, our Taiji pipeline predicted TFs as being selectively active in either of these two cell states. This could aid in developing better immunotherapies, in which one can engineer T cells away from exhaustion and towards more functional effector cell states without negatively affecting T_RM_ cell formation in tissues and tumours. On the basis of statistical criteria (Extended Data Fig. 2a), we identified 20 and 34 TFs as single-state TFs of T_RM_ and TEX_term_ cells, respectively, and 30 multi-state TFs that were active in both (Fig. 1f–h, Extended Data Fig. 2a (blue boxes) and Supplementary Table 3). TEX_term_ single-state TF genes included those for many previously unreported TFs, such as *Zscan20*, *Jdp2*, *Zfp324*, *Zfp143*, *Zbtb49* and *Arid3a* (Fig. 1f). T_RM_ single-state TF genes included *Fosb*, *Zfp692*, *Atf4*, *Pbx4*, *Junb* and *Klf6* (Fig. 1g). Of the TEX_term_ and T_RM_ multi-state TF genes, some, such as *Nr4a2* (ref. 12), *Bhlhe40* (ref. 23) and *Prdm1* (refs. 22,31), were well known to function in the development of both cell states, whereas others, such as *Hic1* (ref. 38) and *Gfi1* (ref. 39), were not, identifying them as new multi-state TFs to consider (Fig. 1h). We analysed previously reported TFs such as cJUN, BATF/BATF3 and TFAP4 that were identified from functional screening of CD8^+^ T cells^40–43^ based on limited phenotypic readouts. These previous screens tended to identify broadly active, multi-state TFs (Fig. 1). By contrast, our platform enabled a computationally guided, multi-state screen that identified TFs predicted to have greater state-selective activity (Extended Data Fig. 2d).

**Extended Data Fig. 3 |:**
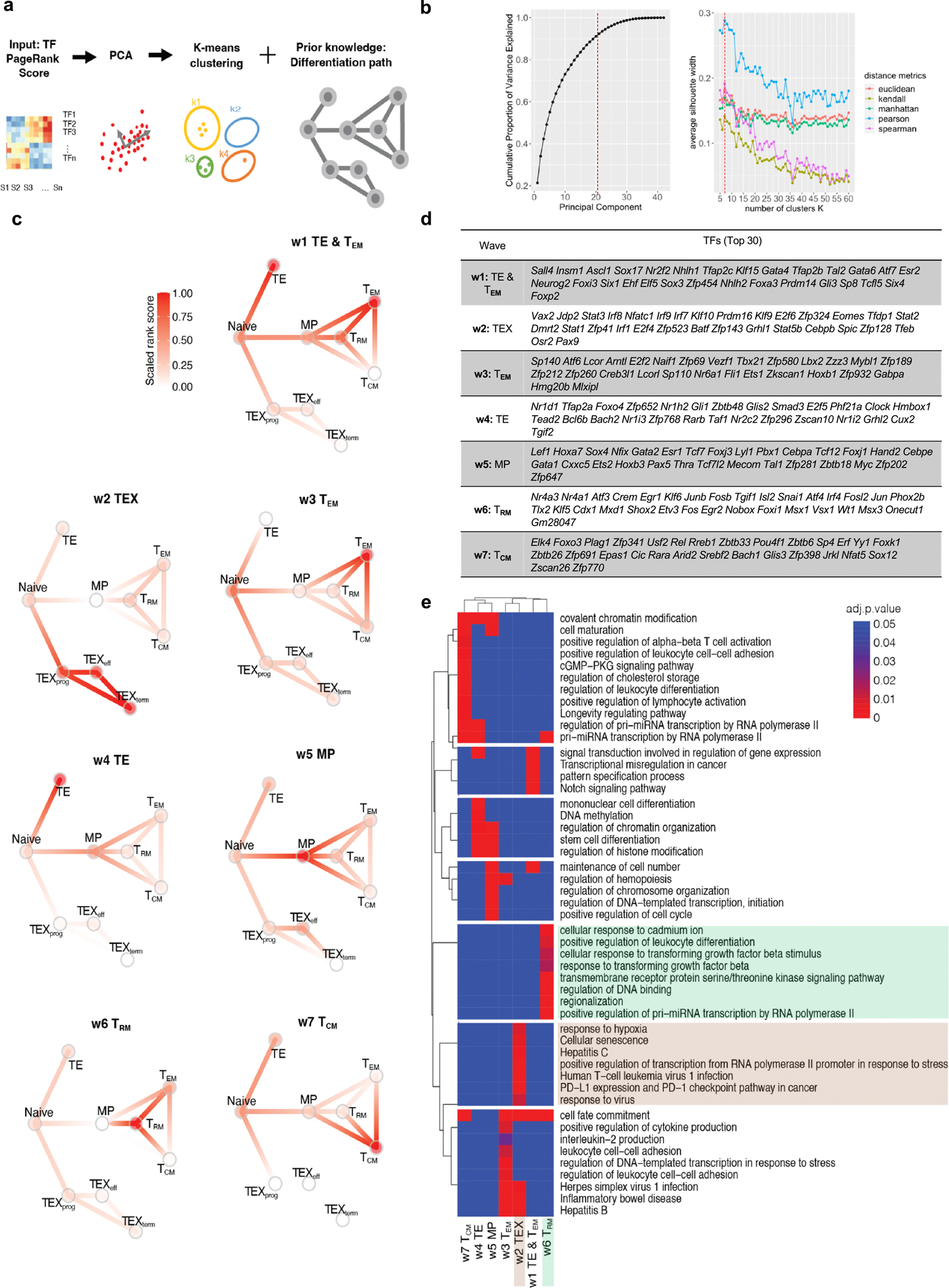
TF wave analysis. **a**, Schematic of the analysis pipeline. **b**, Selection of algorithms and parameters for TF wave analysis. The Pearson correlation was chosen for the distance metric, with k = 7 chosen as the optimal cluster number. **c**, Seven TF waves. Circles represent specific cell states. Red color indicates normalized PageRank scores. **d**, List of TF members in each wave. **e**, Heatmap of biological pathways enriched in each TF wave. Red-blue color scale indicates the p-value.

**Extended Data Fig. 4 |:**
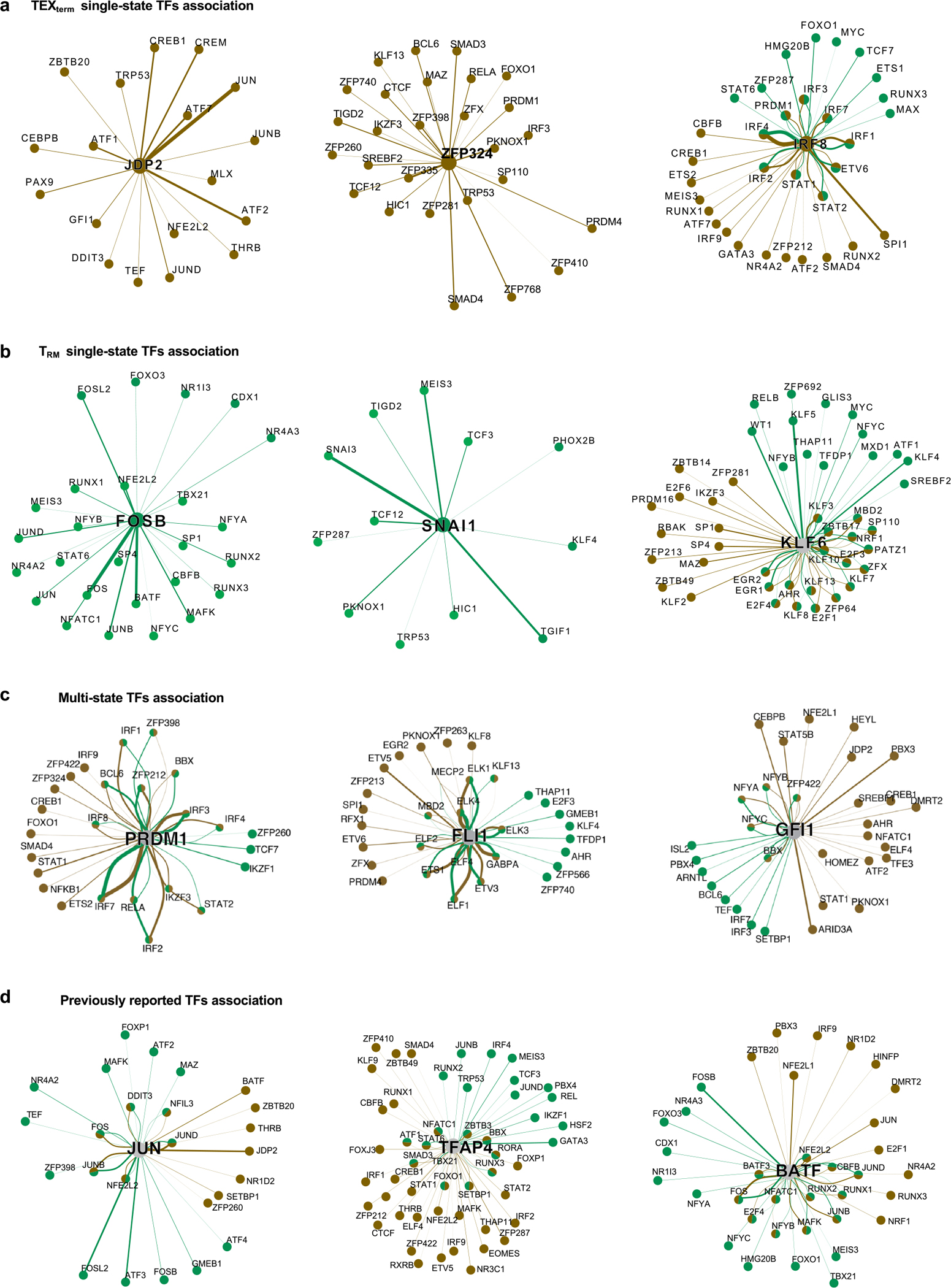
TF–TF association network in TEX_term_ and T_RM_ cell states. **a–c**, TF–TF associations of (**a**) TEX_term_ single-state TFs (JDP2, ZFP324, and IRF8); (**b**) T_RM_ single-state TFs (FOSB, SNAI1, and KLF6); (**c**) multi-state TFs shared by TEX_term_ and T_RM_ (PRDM1, FLI1, and GFI1); and (**d**) previously reported TFs whose OE prevent TEXterm–JUN, TFAP4, and BATF. Line color indicates state specificity: T_RM_ (green) or TEX_term_ (brown) state. Line thickness represents interaction intensity. Line color indicates state specificity: T_RM_ (green) or TEX_term_ (brown) state. Line thickness represents interaction intensity.

To evaluate the TFs that were predicted to govern selective T cell differentiation, we identified dynamic activity patterns of TF groups, termed ‘TF waves’ (Extended Data Fig. 3). TF waves reveal possible combinations of TFs that coordinate trajectories. Seven TF waves linked to specific biological pathways were identified, such as the T_RM_ TF wave (Fig. 1i), which includes genes encoding several members of the AP-1 family (for example, *Atf3*, *Fosb* and *Jun*) that are associated uniquely with the TGFβ response pathway (Extended Data Fig. 4e). The TEX TF wave, which involves *Irf8*, *Jdp2*, *Nfatc1* and *Vax2*, correlates with PD1 and senescence pathways (Fig. 1j and Extended Data Fig. 3e).

## TF community analysis of T_RM_ versus TEX_term_ cells

**Fig. 2 |:**
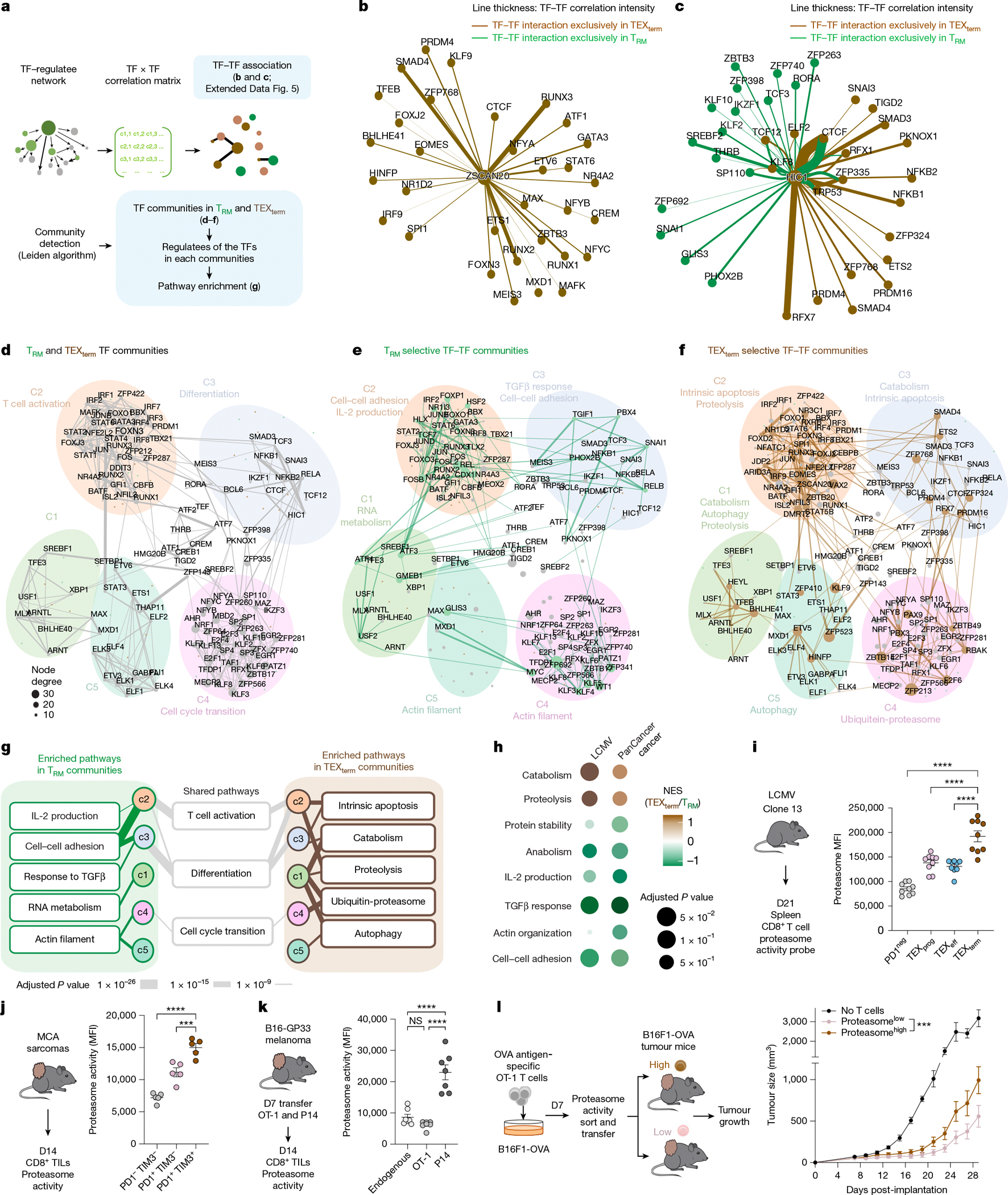
Global analysis of TF networks in TEX_term_ and T_RM_ cell states. **a**, Overview of TF–TF network analysis encompassing association and community-level organization of T_RM_ and TEX_term_ regulatory landscapes. **b**,**c**, TF–TF association networks focused on the TEX_term_ single-state TF ZSCAN20 (**b**) and the multi-state TF HIC1 (**c**), depicting predicted context-specific interactions in T_RM_ (green) or TEX_term_ (brown) cells. **d**–**f**, Clustering of TF–TF associations identified five distinct TF communities in T_RM_ and TEX_term_ networks. Shared TFs (grey) shape overall community topology (**d**), whereas T_RM_- or TEX_term_-specific interactions are represented as green (**e**) or brown (**f**) edges, respectively. **g**, Summary of shared and unique biological pathways enriched within T_RM_ and TEX_term_ communities. Line thickness reflects −log_10_ (*P* value). Pathway gene sets in Supplementary Table 8. **h**, Gene set enrichment analysis (GSEA) comparing TEX_term_ versus T_RM_ cell pathways using batch-effect corrected LCMV bulk RNA-seq^3,9,17,22,31–35^ and human pan-cancer scRNA-seq data sets^44,55,61^. **i**–**k**, Flow cytometry analysis of proteasome activity showing the highest activity in TEX_term_ cells during LCMV–Clone-13 infection (**i**) and MCA-205 tumours (**j**). In dual transfer experiments, antigen-specific (P14) and bystander (OT-1) CD8^+^ T cells analysed from B16-GP33 tumours (**k**) show elevated proteasome activity in TEX_term_-like populations. **l**, Functional impact of proteasome activity on tumour growth. Tumour-bearing C57BL/6 mice were infused with proteasome^high^ or proteasome^low^ OT-1 cells pre-stimulated with B16F1-OVA tumour cells for 7 days. Proteasome^high^ OT-1 cells exhibit reduced tumour control. Data are shown as mean ± s.e.m. Ordinary one-way analysis of variance (ANOVA) (**i**–**k**) and two-way ANOVA Tukey’s multiple comparison test (**l**) were performed. **i**–**l**, *n* ≥ 6. ****P < 0.0001, ***P < 0.001, **P < 0.01, *P < 0.05.

To uncover transcriptional programmes governing T_RM_ or TEX_term_ cell differentiation, we constructed TF–TF association networks capturing functional relationships between TFs (Fig. 2a). Analysis of regulatee-based adjacency matrices (that is, predicted TF–target gene circuits) revealed shared and distinct patterns of TF collaboration across the two states. Single-state TFs displayed strong intra-state connectivity. TEX_term_ TFs (ZSCAN20, JDP2, ZFP324, IRF8) formed dense networks within TEX_term_ cells (Fig. 2b and Extended Data Fig. 4a), whereas T_RM_ TFs (FOSB, SNAI1, KLF6) interacted mainly within T_RM_ networks (Extended Data Fig. 4b). Multi-state TFs (HIC1, PRDM1, FLI1, GFI1) that were active in both states and previously reported TFs (cJUN, BATF and TFAP4) formed distinct partnerships in each cell state, reflecting context-specific regulatory architectures (Fig. 2c and Extended Data Fig. 4c).

We next grouped the TF–TF association networks into distinct ‘TF neighbour communities’ in T_RM_ and TEX_term_ cells (Supplementary Table 5), and each community was linked to specific biological processes (Fig. 2d–f). Although multi-state TFs shaped overall community topology, single-state TFs drove unique interaction patterns specific to T_RM_ or TEX_term_ cells within each community. Pathway analysis revealed divergent programmes in each state—for instance, T_RM_ community-3 was associated with cell adhesion and TGFβ response (Fig. 2e,g,h), whereas TEX_term_ community-3 was linked to apoptosis (Fig. 2f–h). Community-1 in T_RM_ cells controlled RNA metabolism (Fig. 2e,g,h), whereas in TEX_term_ cells, it was tied to catabolism, proteolysis and autophagy (Fig. 2f–h).

**Extended Data Fig. 5 |:**
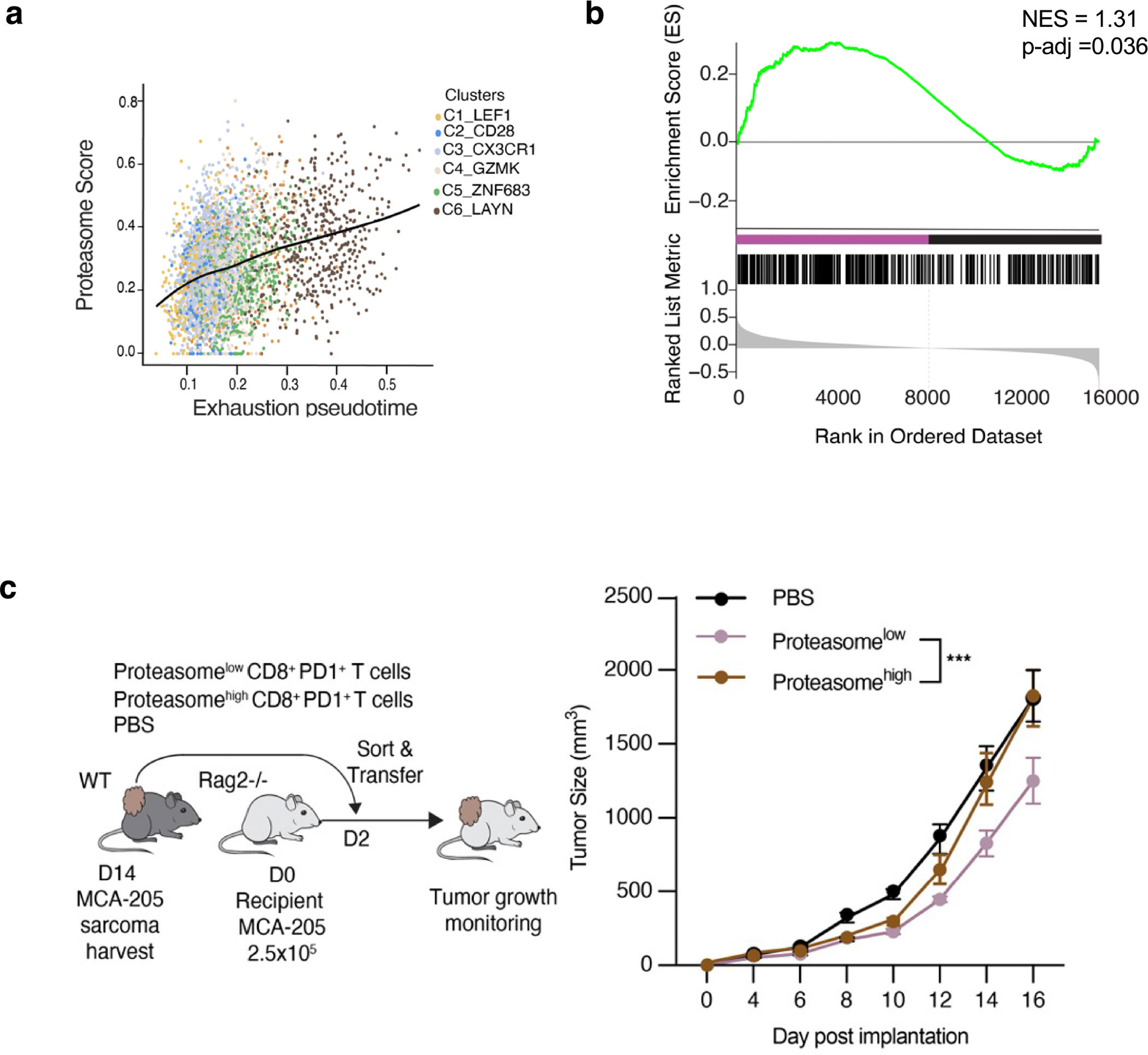
TF network analysis reveals proteasome pathway enrichment in TEX_term_ state with diminished tumor control function. **a**, Pseudotime analysis of CD8^+^ TIL scRNA-seq data from NSCLC patients (n = 14), showing a positive correlation between proteasome gene scores (KEGG: M10680) and T cell exhaustion. **b**, Gene set enrichment analysis (GO:0043161, Proteasome-mediated ubiquitin-dependent protein catabolic process) of RNA-seq from CD8^+^ splenocytes (black) and TILs (purple), n = 3 each. **c**, Tumor growth of Rag2^−/−^ mice bearing MCA-205 sarcomas infused with proteasome^high^ or proteasome^low^ CD8^+^ TILs isolated from C57BL/6 mice bearing MCA-205 tumors. n = 5 per group. Two-way ANOVA Tukey’s multiple comparison test were performed. ****P < 0.0001, ***P < 0.001, **P < 0.01, *P < 0.05.

To assess the functional relevance of state-enriched pathways, we focused on the proteasome pathway, which emerged as a prominent but previously unrecognized feature of TEX_term_ cells (Fig. 2g,h). Proteasome gene signatures were enriched in TEX_term_-like CD8^+^ T cells from patients with non-small cell lung cancer (NSCLC)^44^ and mouse MCA-205 TILs (Extended Data Fig. 5a,b). Consistently, proteasome activity—measured by a validated fluorescent probe^45^—was highest in TEX_term_ cells from chronic LCMV (Fig. 2i) and in tumour-specific TILs (Fig. 2j,k) relative to bystander OT-1 cells (Fig. 2k). To test whether high proteasome activity correlates with dysfunction, we sorted OT-1 cells by proteasome activity probe intensity and adoptively transferred them into B16F10-OVA tumour-bearing mice. Proteasome^high^ cells showed reduced tumour control compared with proteasome^low^ cells (Fig. 2l)—a trend also seen in endogenous TILs (Extended Data Fig. 5c). These findings support the TF–TF network and pathway predictions and identify the proteasome pathway as a functional hallmark of TEX_term_ cells.

## In vivo CRISPR screens of TEX_term_ TFs

**Fig. 3 |:**
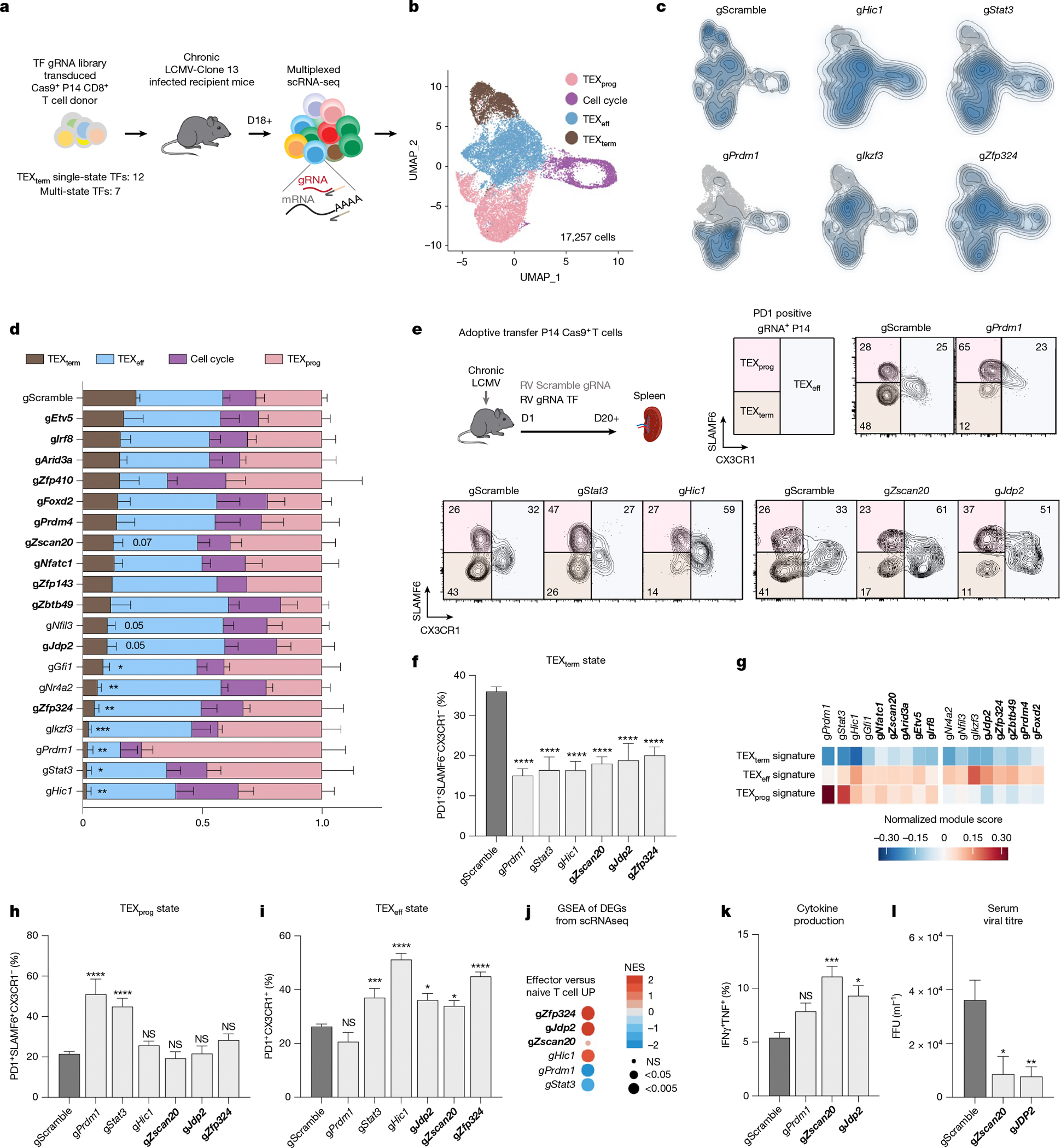
In vivo Perturb-seq validation of TEX_term_-driving TFs. **a**, Schematic of the in vivo Perturb-seq strategy. Cas9^+^P14^+^ TCR transgenic CD8^+^ T cells recognizing the LCMV epitope GP33–41 were transduced with retrovirus-expressing gRNA libraries, adoptively transferred into mice infected previously (1 day earlier) with LCMV–Clone-13, and analysed 18–23 days later by scRNA-seq. **b**, UMAP showing TEX_prog_, TEX_eff_, TEX_term_ and cell cycle clusters; marker expression is in Extended Data Fig. 6b,c. **c**,**d**, Kernel density plots (**c**) and distributions of gRNA^+^ cells (**d**) across clusters. TEX_term_ single-state TF genes are in bold. Data represent five pooled replicates from three independent experiments; values are shown as mean ± s.e.m. Statistical analysis: two-way ANOVA with Fisher’s least significant difference (LSD) test compared with the gScramble control; results for TEX_term_ and TEX_prog_ clusters are shown; with full comparisons in Supplementary Table 7; ****P < 0.0001, ***P < 0.001, **P < 0.01, *P < 0.05. **e**, Representative flowplots showing phenotyping of the TF KOs in LCMV–Clone 13-infected mice. **f**, Quantification of TEX_term_ (PD1^+^SLAMF6^−^CX3CR1^−^) frequencies in donor CD8^+^ T cells. **g**, Differential expression analysis of TEX_term_, TEX_prog_ and TEX_eff_ gene signatures^46^ (Supplementary Table 8) across each TF KO. **h**,**i**, Frequencies of TEX_prog_ (PD1^+^SLAMF6^+^CX3CR1^−^) (**h**) and TEX_eff_ (PD1^+^CX3CR1^+^) (**i**) subsets. **j**, GSEA showing enrichment of effector-associated gene sets in TF KOs versus control. **k**,**l**, Functional validation: cytokine production (IFNγ, TNF) and viral titres in mice receiving TF KO versus control CD8^+^ T cells. Statistical analysis for **f**, **h**, **i**, **k**, **l,** mean ± s.e.m., ordinary one-way ANOVA with Dunnett’s multiple comparison versus gScramble (**f**–**k**, *n* ≥ 8, at least three biological replicates; **i**, *n* ≥ 4, at least two biological replicates). ***P < 0.001, **P < 0.01, *P < 0.05.

**Fig. 4 |:**
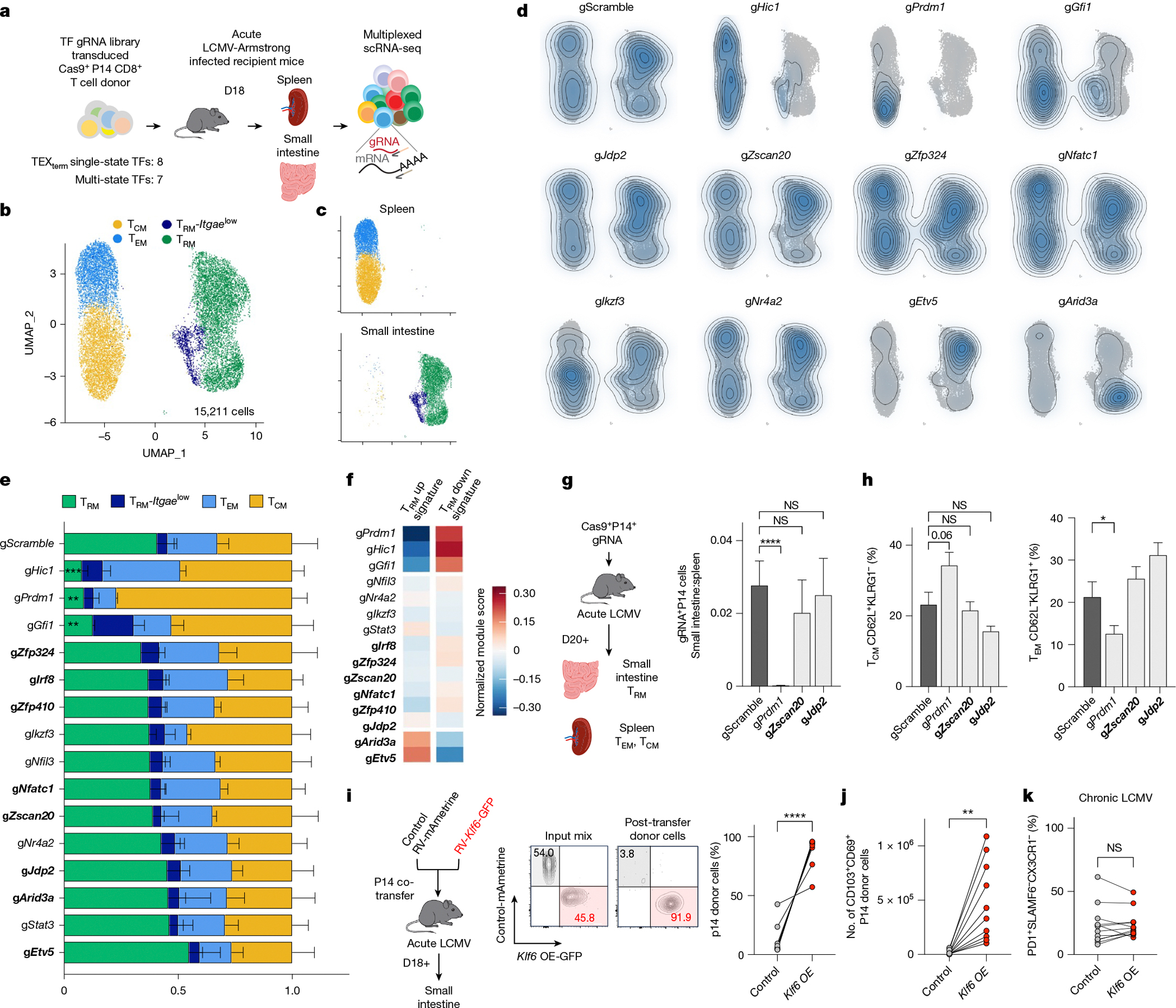
Functional validation of TFs with distinct roles in TEX_term_ and T_RM_ differentiation. **a**, Schematic of in vivo Perturb-seq screening during acute LCMV–Armstrong infection to assess memory CD8^+^ T cell differentiation. Transduced donor Cas9^+^P14^+^CD8^+^ T cells were analysed for T_RM_, T_EM_ and T_CM_ states in the small intestine and spleen. **b**, UMAP embedding of 15,211 cells identifying T_CM_ (*Il7r*, *Tcf7*, *Sell*, *S1pr1*), T_EM_ (*Cx3cr1*, *Klrg1*, *Klf2*), T_RM_ (*Cd69*, *Itgae*, *Cd160*) and T_RM_- *Itgae*^low^ clusters. **c**, Differential distribution of cells across tissues. **d**, Kernel density map of gRNA^+^ cells in UMAP space. **e**, Cluster distribution for each TF gRNA; TEX_term_ single-state TF genes are in bold. Data represent three replicates; mean ± s.e.m. Statistical analysis, two-way ANOVA with Fisher’s LSD versus gScramble. ****P < 0.0001, ***P < 0.001, **P < 0.01, *P < 0.05; Supplementary Table 5. **f**, Normalized expression of T_RM_ ‘up’ and ‘down’ gene signatures^9,31^ in each KO versus control. **g**,**h**, Phenotypic validation. Ratio of gRNA^+^ cells in small intestine to spleen (**g**) and frequency of splenic T_CM_ (CD62L^+^KLRG1^−^) and T_EM_ (CD62L^−^KLRG1^+^) cells (**h**). **i**,**j**, Overexpression (OE) of the T_RM_ single-state TF gene *Klf6* enhances T_RM_ formation. P14^+^CD8^+^ T cells transduced with *Klf6* or empty vector were co-transferred (approximately 1:1) into LCMV–Armstrong-infected mice. **i**, Representative plots pre- and post-transfer. **j**, Quantification of donor CD69^+^CD103^+^ T_RM_ cells in the small intestine. **k**, Quantification of the frequency of TEX_term_ cells. Statistical tests, ordinary one-way ANOVA with Dunnett’s multiple comparison versus gScramble (**g**,**h**), paired *t*-tests (**i**,**j**); *n* ≥ 4 (**g**,**h**) or *n* ≥ 6 (**i**–**k**) from at least two biological replicates. Data, mean ± s.e.m. ***P < 0.001, **P < 0.01, *P < 0.05.

The Taiji pipeline enabled comparative analysis of TF activity and curated sets of single-state TFs specific to T_RM_ versus TEX_term_ cells (Fig. 1f–h). To assess its accuracy, Perturb-seq, combining in vivo CRISPR screening with single-cell RNA-seq (scRNA-seq), was performed in two animal models for T_RM_ or TEX_term_ differentiation (Figs. 3a and 4a). Our Perturb-seq guide RNA (gRNA) library targeted 19 TF genes, including 7 encoding TEX_term_ and T_RM_ multi-state TFs and 12 encoding TEX_term_ single-state TFs. The TEX_term_ TF genes included one known TF (*Nfatc1*) and 11 others that had high specificity scores but were not previously linked to TEX_term_ differentiation (grey boxes; Fig. 1f,h). The multi-state TF genes included two positive controls (*Nr4a2*, *Prdm1*) and unvalidated multi-state TF genes (*Nfil3*, *Hic1*, *Gfi1*, *Ikzf3*, *Stat3*). To ensure comprehensive screening, four gRNAs per target were expressed in two dual-gRNA retroviral vectors (Extended Data Fig. 4a), along with two control vectors with scramble gRNAs (gScramble). This created a library of 40 dual-gRNA vectors, with 76 TF-gRNAs and four gScramble controls (Supplementary Table 6).

Cas9^+^ P14 CD8^+^ T cells were transduced with this library and transferred into mice infected with LCMV–Clone-13—a model of chronic infection and CD8^+^ T cell exhaustion (recipient mice also expressed Cas9 to prevent rejection of donor cells). Droplet-based sequencing was performed 18 or more days post-transfer to assess sgRNA and transcriptomes of each spleen-derived donor Cas9^+^ P14 CD8^+^ T cell (Fig. 3a), analysing 17,257 cells with unique gRNA expression.

To determine which TF genes impaired TEX_term_ cell differentiation, we first used uniform manifold approximation and projection (UMAP). Four primary clusters were identified: TEX_prog_, TEX_eff_ and TEX_term_ cells and those in cell cycle (Fig. 3b and Extended Data Fig. 4b,c). All clusters expressed *Tox* and *Pdcd1*—key exhaustion markers—and TEX_prog_ cells were identified by *Tcf7*, *Slamf6* and *Sell* expression. TEX_eff_ cells expressed effector markers, including *Cx3cr1*, *Klrd1*, *Klrk1*, *Klf2* and *Zeb2* (ref. 2), whereas TEX_term_ cells expressed high inhibitory receptors and well-established exhaustion markers such as *Cd101*, *Cd7*, *Cd38*, *Cd39*, *Cxcr6* and *Nr4a2*. The cell cycle cluster was noted for its expression of *Birc5*, *Mki67*, *Stmn1* and *Tuba1b*.

Next, we evaluated the impact of individual TF depletion by analysing the distribution of gRNA^+^ cells across exhaustion states (Fig. 3c,d). CRISPR knockout (KO) of most of the 19 TEX_term_-driving TF genes led to a reduction in TEX_term_ cell frequency. Notably, KOs of multi-state TF genes such as *Hic1*, *Stat3*, *Prdm1* and *Ikzf3* (which encodes AIOLOS) resulted in a profound reduction of approximately 90% in TEX_term_ differentiation. Depletion of new TEX_term_ single-state TF genes—including *Zfp324*, *Zscan20* and *Jdp2*—reduced TEX_term_ differentiation significantly, by 78%, 54% and 43%, respectively (Fig. 3d, bold). Other new candidates, such as *Etv5*, *Arid3a*, *Zfp410*, *Foxd2* and *Prdm4*, also reduced TEX_term_ representation by 25–40%, although some did not reach statistical significance. This Perturb-seq analysis highlights the platform’s ability to identify TFs that regulate the TEX_term_ state, with most tested TFs influencing exhaustion to varying degrees.

To further assess how KO of TEX_term_-driving TF genes affect CD8^+^ T cell exhaustion, we used flow cytometry and scRNA-seq to analyse TF KO cells during LCMV–Clone 13 infection (Fig. 3e–i and Extended Data Fig. 4). We tested six TF KOs, including known control (*Prdm1*) and five newly identified TF genes (*Zscan20*, *Jdp2*, *Zfp324*, *Stat3*, *Hic1*) that impaired TEX_term_ state differentiation in Perturb-seq. Disrupting these TFs reduced TEX_term_ cell (PD1^+^CX3CR1^−^SLAMF6^−^) frequency by around 50% (Fig. 3e,f) and decreased expression of inhibitory receptors such as CD101, CD39 and CD38 (Extended Data Fig. 4d,e). All 19 TEX_term_-TF gene KOs exhibited a marked decrease in TEX_term_-signature genes^46^, including *Cd7*, *Cxcr6*, *Nr4a2* and *Entpd1* (Fig. 3g).

Finally, the TEX_term_-driving TF gene KOs were grouped according to their effects on TEX_prog_ (PD1^+^CX3CR1^−^SLAMF6^+^; Fig. 3h) or TEX_eff_ (PD1^+^CX3CR1^+^; Fig. 3i) state differentiation. Loss of *Prdm1* and *Stat3* markedly increased the frequency of TEX_prog_ cells and upregulated TEX_prog_ signature genes (Fig. 3g,h) whereas loss of *Hic1*, *Zscan20*, *Zfp324* or *Jdp2* expanded primarily the TEX_eff_ cell population and effector signature genes (Fig. 3g,i,j). Deletion of the *Zscan20* and *Jdp2* significantly enhanced effector cytokine production (for example, interferon gamma (IFNγ) and tumour necrosis factor (TNF)) and reduced viral loads in recipient mice (Fig. 3k,l).

## Deleting TEX_term_ TFs preserves T_RM_ fate

A principal goal of this work was to identify TFs that selectively repress TEX_term_ cell differentiation without affecting T_RM_ differentiation, thereby enabling more precise programming of CD8^+^ T cell states. As nearly all the predicted TEX_term_ single-state TFs impaired TEX_term_ differentiation to some degree (Fig. 3), the next step was to evaluate their effects on T_RM_ differentiation to confirm their selective activity. We used the same Perturb-seq library as before, but this time included only the eight TEX_term_ single-state TFs and seven multi-state TFs that impaired TEX_term_ state development by more than 25% in chronic LCMV infection (Fig. 3d). To assess their impact on memory CD8^+^ T cell development, we isolated retrovirus-transduced Cas9^+^ P14 CD8^+^ T cells from the spleen and small intestine of mice 18 days after acute LCMV–Armstrong infection. We then analysed 15,211 cells using scRNA-seq to determine how these perturbations affected the formation of intestinal T_RM_ cells, as well as circulating splenic T_CM_ and T_EM_ cells (Fig. 4a).

The UMAP analysis identified four primary clusters containing cells with features of T_CM_ (*Il7r*, *Tcf7*, *Sell* and *S1pr1*), T_EM_ (*Cx3cr1*, *Klrg1* and *Klf2*) and T_RM_ cells (*Cd69*, *Cd160* and *Itgae* (encoding CD103)) as well as a small T_RM_ cell population with lower *Itgae* but higher *Ifng* and *Irf1* expression designated T_RM_-*Itgae*^low^ (refs. 22,47) (Fig. 4b,c and Extended Data Fig. 7a,b). Examination of the gRNA^+^ cells revealed that none of the eight TEX_term_ single-state TF gene KOs (*Zfp324*, *Irf8*, *Zfp410*, *Nfatc1*, *Zscan20*, *Jdp2*, *Arid3a* and *Etv5*) negatively affected T_RM_ formation significantly (bold gene names in Fig. 4d,e). In fact, KO of *Etv5* tended to increase the frequency of T_RM_ cells. To evaluate the specificity of the TEX_term_ single-state TF genes, we also examined the expression of the T_RM_ gene signatures^9,31^ in the entire population of gRNA^+^ cells for each TF tested (Fig. 4f). With the exception of *Etv5* and *Arid3a*, KO of which increased T_RM_-signature gene expression (Fig. 4f and Extended Data Fig. 7c), perturbation of the TEX_term_ single-state TFs did not substantially alter T_RM_-signature gene expression. The platform also predicted new multi-state TFs, including those encoded by *Hic1* and *Gfi1*. Disruption of these multi-state TFs significantly reduced T_RM_ cell frequency (Fig. 4e) and T_RM_-signature gene expression (Fig. 4f and Extended Data Fig. 7c), mirroring the effects of disruption of *Prdm1*, which encodes a known multi-state TF for T_RM_ and TEX_term_ cells.

To further validate the Perturb-seq data, we depleted the TEX_term_ single-state TF genes *Zscan20* and *Jdp2* and the multi-state TF gene *Prdm1* individually in Cas9^+^ P14 CD8^+^ T cells, transferred them adoptively into LCMV–Armstrong infected animals, and assessed their differentiation into T_CM_, T_EM_ and T_RM_ cells using flow cytometry (Fig. 4g,h). Deletion of *Zscan20* and *Jdp2* did not alter the formation of any memory cell subtypes, whereas perturbation of *Prdm1* reduced T_RM_ and increased T_CM_ formation significantly, as expected. Altogether, this multi-omics pipeline predicted TEX_term_ single-state TFs that drive TEX_term_ differentiation without affecting T_RM_ cell formation and multi-state TFs that influence both cell states. These results demonstrate the accuracy and predictive power of our approach for pinpointing single-state and multi-state TFs.

## *Klf6* overexpression expands T_RM_ cells without exhaustion

To further demonstrate the utility of our cell-state selective TF identification pipeline in discovering new T_RM_-associated TFs, we evaluated *Klf6*, which was identified through our Taiji analysis as a T_RM_ single-state TF gene (Fig. 1g). We considered whether overexpressing *Klf6* (*Klf6*-OE) would enhance T_RM_ formation during acute viral infection without worsening terminal exhaustion in chronic infection. Our results confirmed this suggestion. When empty-vector control and *Klf6-*OE P14 CD8^+^ T cells were co-transferred, *Klf6-*OE cells robustly outcompeted control cells, resulting in 15-fold enrichment in the small intestine compared with controls (Fig. 4i). Furthermore, there werearound 42 times more CD69^+^CD103^+^ double-positive T_RM_-like cells in *Klf6-*OE than in control donor cells, indicating that *Klf6*-OE markedly increased T_RM_ development in the small intestine (Fig. 4j). *Klf6-*OE did not increase terminal exhaustion during chronic infection (Fig. 4k and Extended Data Fig. 7d). This work not only identifies KLF6 as a new T_RM_-driving TF but also confirms its selectivity.

## New TEX_term_-TF loss improves tumour control

**Fig. 5 |:**
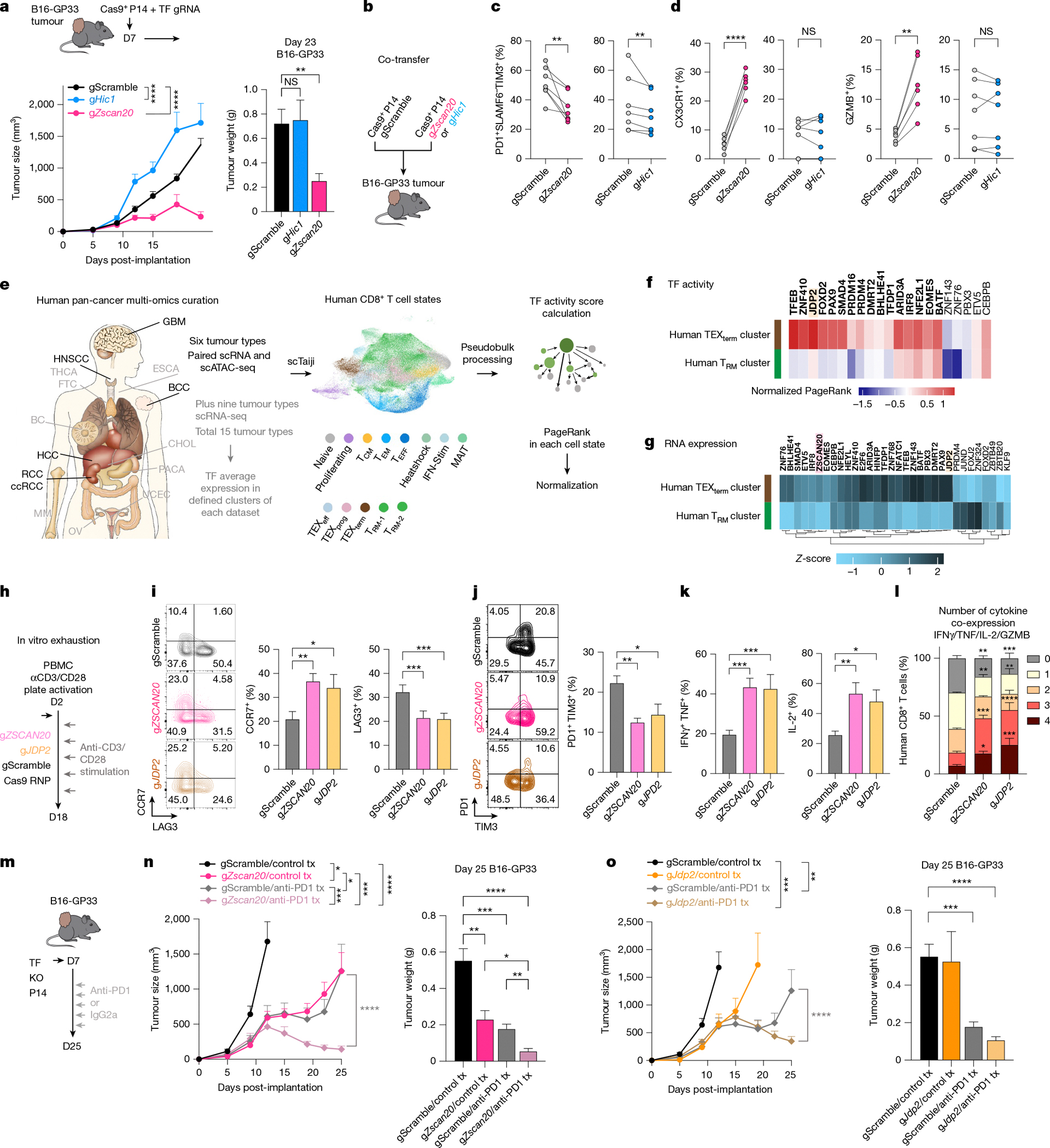
Targeting TEX_term_ single-state TFs enhances tumour control. **a**, Experimental design and tumour outcomes from adoptive transfer of P14 CD8^+^ T cells carrying CRISPR KOs of *Zscan20* (TEX_term_ single-state TF gene) or *Hic1* (multi-state TF gene active in TEX_term_ and T_RM_ cells) into B16-GP33 melanoma-bearing mice. Tumour volumes and terminal weights are shown. **b**, Co-transfer design mixing *Zscan20*-KO or *Hic1*-KO Cas9^+^ P14 cells with scramble controls before transfer. **c**,**d**, Quantification of PD1^+^SLAMF6^−^TIM3^+^ exhausted subsets (**c**) and CX3CR1^+^ and GZMB^+^ effector populations (**d**) in *Zscan20*-KO and *Hic1*-KO cells. **e**, Human pan-cancer single-cell multi-omics and scRNA-seq datasets^48–55^ were integrated to assess TF expression and activity across CD8^+^ T cell states using scTaiji. BC, breast cancer; CHOL, cholangiocarcinoma; ESCA, oesophageal cancer; FTC, follicular thyroid cancer; MM, multiple myeloma; OV, ovarian cancer; PACA, pancreatic cancer; THCA, thyroid cancer; UCEC, uterine corpus endometrial carcinoma. **f**, Paired scRNA-seq and scATAC-seq were used to build regulatory networks and compute PageRank TF activity scores. Shown are normalized scores for TEX_term_ single-state TF genes (Fig. 1f) with conserved DNA-binding motifs in humans. **g**, mRNA expression of TEX_term_ TF genes across TEX_term_ and T_RM_ clusters in human tumours; cross-species conserved TF genes are in bold. **h**, Human peripheral blood mononuclear cell (PMBC) KO design. *ZSCAN20*-KO or *JDP2*-KO CD8^+^ T cells were stimulated with anti-CD3/CD28 beads for 18 days to model chronic activation. **i**,**j**, Flow cytometry analysis of CCR7 (memory-like and stem-like) (**i**) and the inhibitory receptors LAG3, PD1 and TIM3 (**j**) in KO versus control cells. **k**, Frequencies of IFNγ^+^TNF^+^ and interleukin-2 (IL-2)^+^ cells. **l**, Polyfunctionality analysis of cytokine-producing cells. **m**, Schematic of adoptive transfer and anti-PD1 treatment testing synergy with TEX_term_ TF gene KO. Cas9^+^ P14 cells (±TF KO) were transferred into B16-GP33 tumours and treated with anti-PD1 or IgG2a. D7, day 7; D25, day 25. **n**,**o**, Tumour growth and weights for *Zscan20*-KO (**n**) and *Jdp2*-KO (**o**) versus controls. Data are mean ± s.e.m.; *n* ≥ 6 from at least two biological replicates. Statistics, two-way ANOVA with Tukey’s (tumour volume in **a**, **n**, **o**); one-way ANOVA with Dunnett’s (**i**–**k,** tumour weights in **a**, **n**, **o**); paired *t*-tests (**c**, **d**); two-way ANOVA with Dunnett’s (**l**). ****P < 0.0001, ***P < 0.001, **P < 0.01, *P < 0.05.

This platform predicted cell-state-selective TF activity and identified TEX_term_ single-state TFs as targets for engineering T cells that resist exhaustion yet retain effector and memory functions—offering new strategies to improve immunotherapy efficacy. Given that T_RM_ cells are associated with better clinical outcomes in solid tumours^9–12^, we hypothesized that KO of exhaustion-selective TF genes such as *Zscan20* could be more effective than targeting T_RM_ and TEX_term_ multi-state TF genes such as *Hic1*. Using an ACT model, we transferred TF gRNA retrovirus-transduced Cas9^+^ P14 CD8^+^ T cells into mice with established melanoma tumours expressing GP33–41 (Fig. 5a). Unlike depletion of the multi-state TF gene *Hic1*, depleting the TEX_term_ single-state TF gene *Zscan20* resulted in improved tumour control (Fig. 5a). Moreover, *Zscan20* gRNA^+^ cells more readily formed TEX_prog_ cells than TIM3^+^ or CD39^+^ TEX_term_ cells (Extended Data Fig. 8a–d). To control for inter-mouse variability in antigen load, we co-transferred *Zscan20* or *Hic1* KO cells with control P14 CD8^+^ T cells into the same B16-GP33 tumour-bearing mice (Fig. 5b–d and Extended Data Fig. 8e–i). Both KOs significantly increased the frequency of PD1^+^SLAMF6^+^TIM3^−^ cells and decreased the frequency of TIM3^+^ exhausted cells and the TEX_term_ cell state (PD1^+^SLAMF6^−^CX3CR1^−^) compared with controls (Fig. 5c and Extended Data Fig. 8f–h), consistent with their predicted activity in TEX_term_ cells (Fig. 1f,h). However, *Zscan20* KO robustly enhanced effector marker expression (CX3CR1), granzyme B and cytokine production in TILs, whereas *Hic1* KO did not seem to improve effector function to the same degree (Fig. 5d and Extended Data Fig. 8i). Thus, despite their similar effects on suppressing TEX_term_ cell differentiation in tumours, differences in their ability to promote functional effector-like states may underlie the differential tumour control observed. Given that HIC1 functions as a multi-state TF and ZSCAN20 as a single-state TF, these findings support the general rationale for targeting state-specific TFs to enable more selective programming of T cell differentiation.

## State-selective TFs conserved across species

To evaluate the relevance of our mouse findings in human T cells—particularly for applications in immunotherapy—we conducted cross-species validation using publicly available single-cell multi-omics and scRNA-seq datasets from human tumour-infiltrating CD8^+^ T cells (Fig. 5e, Extended Data Fig. 9a and Supplementary Table 1). Leveraging the Taiji TF analysis platform, we mapped mouse TEX_term_-associated and T_RM_-associated TF genes onto a curated human pan-cancer CD8^+^ T cell atlas encompassing six tumour types^48–55^ (glioblastoma (GBM), head and neck squamous cell carcinoma (HNSCC), basal cell carcinoma (BCC), hepatocellular carcinoma (HCC), renal cell carcinoma (RCC) and clear cell renal cell carcinoma (ccRCC)). Human CD8^+^ T cells were clustered into heterogeneous cell states, including T_RM_ and TEX_term_ clusters (Fig. 5e and Extended Data Fig. 9b). Taiji analysis revealed strong cross-species conservation: TEX_term_ TF genes such as *JDP2*, *ZNF410* and *FOXD2* exhibited higher activity in TEX_term_ clusters than in T_RM_-like cells (Fig. 5f). Of 34 mouse TEX_term_ single-state TF genes, 19 showed conserved activity patterns in human TEX_term_ cells. Similarly, T_RM_-specific TF genes (for example, *NR4A1*, *KLF6* and *FOSB*) displayed enriched activity in the human T_RM_ cluster (Extended Data Fig. 9c). Furthermore, 22 of the 30 mouse TF genes that were active in both TEX_term_ and T_RM_ states showed similar activity profiles in human datasets (Extended Data Fig. 9d). A few TF genes—such as *ZSCAN20*—could not be assessed in the Taiji analysis because of missing DNA-binding motifs, but comparative RNA profiling across 15 tumour types supported their relevance, with 24 of 34 mouse TEX_term_ single-state TF genes, including *ZSCAN20* and *JDP2*, showing higher expression in human TEX cells (Fig. 5g).

Given these correlations between species, we perturbed *ZSCAN20* and *JDP2* to assess the relevance of TEX_term_ single-state TFs in human T cells (Extended Data Fig. 10a,b). Following repeated CD3/CD28 stimulation over 18 days to simulate chronic activation (Fig. 5h), *ZSCAN20*- or *JDP2*-deficient CD8^+^ T cells exhibited increased expression of CCR7 (naive/stem cell memory/T_CM_ marker) and decreased levels of inhibitory receptors, including LAG3, PD1 and TIM3 (Fig. 5i,j). These KO cells also produced higher levels of effector cytokines (Fig. 5k,l), indicating that ZSCAN20 and JDP2 contribute to exhaustion-associated features in human CD8^+^ T cells.

## ICB synergy with *Zscan20* and *Jdp2* KOs

Tumours with high TEX_term_ cell infiltration often exhibit poor responses to ICB therapy^16^. We considered whether targeting TEX_term_ single-state TFs could enhance ICB efficacy. Among the TEX_term_-associated TF genes, *Zscan20* and *Jdp2* were prioritized for their conservation and functional relevance in human T cells (Fig. 5e–l). To test synergy with ICB, treatment began 1 day after adoptive transfer of TF-depleted P14 CD8^+^ T cells (Fig. 5m). The combination of *Zscan20* or *Jdp2*-KO with anti-PD1 therapy significantly reduced tumour burden (Fig. 5n,o) and improved survival (Extended Data Fig. 10c,d). These findings suggest that selectively disrupting TEX_term_ single-state TFs represents a promising strategy to enhance T cell therapy by minimizing dysfunctional states while preserving beneficial T cell phenotypes. Overall, our cross-species multi-omics and functional perturbation approach underscores the translational potential of Taiji-identified TFs for improving ACT.

## Discussion

Our study introduces a powerful platform for identifying TFs that are pivotal in guiding specific CD8^+^ T cell state differentiation during viral infections and tumour progression. Leveraging our comprehensive transcriptional and epigenetic atlas from nine distinct CD8^+^ T cell states, we developed a detailed map of TF activity, creating a unique TF fingerprint for each context. Furthermore, we developed TaijiChat, a web interface for natural language queries of our datasets and literature (Supplementary Methods).

Focusing on two critical cell states TEX_term_ and T_RM_ T cells, we examined similarities and differences of TF activity and their networks in both states and engineered T cells to resist exhaustion while retaining functionality of T_RM_ cells. Using in vivo Perturb-seq, we validated TF activity for TEX_term_ and T_RM_ cells in both acute and chronic infection models. Although recent CRISPR screenings in CD8^+^ T cells have identified TFs that are important in cytotoxicity, memory formation^40–42^, cell enrichment^56^ and exhaustion^57^, a systematic and context-dependent understanding of TF roles across several contexts has been lacking. Our study addresses this gap by generating an accurate catalogue of CD8^+^ T cell state-defining TF genes, enabling cost-effective validation of predicted TF activity and selectivity using Perturb-seq. Furthermore, our study offers broader and new insight into context-dependent TF regulation. Previously, differential TF cooperation in different contexts was reported^25,42,43^. We extend this by analysing global TF associations across cell states, revealing how TF communities regulate T cell-specific pathways, including protein catabolism in T cell exhaustion, which aligns with previous research on protein homoeostasis^45,58,59^. These TF networks reveal how various cellular processes are controlled differentially between T_RM_ and TEX_term_ cells, providing a rationale for their different functional capabilities within tissues.

One of the key outcomes of this study was the identification of new TFs, including ZSCAN20 and JDP2, as TEX_term_ single-state TFs and KLF6 as a T_RM_ single-state TF, and of newly uncovered roles for multi-state TFs such as HIC1 and GFI1. Perturbing TEX_term_ single-state TFs not only prevented T cell exhaustion but also preserved the ability of these cells to differentiate into effector and memory states. This led to significant improvements in tumour control.

To evaluate the clinical importance of the newly discovered TFs and the catalogue of TFs with TEX_term_ and T_RM_ selectivity, we confirmed cross-species conservation of a substantial number of TFs using Taiji analysis of a human pan-cancer multi-omics atlas, along with comparative expression analysis across pan-cancer scRNA-seq datasets. Furthermore, we demonstrated enhanced human T cell function following perturbation of the TEX_term_ single-state TFs ZSCAN20 and JDP2. Depletion of these TFs shows synergistic effects with ICB therapy, leading to significant tumour regression. These findings highlight a promising strategy for enhancing antitumour immunity through precise cell-state programming.

Our TF atlas-guided platform can offer optimized ‘TF recipes’ for cell programming with increased precision, robustness and durability. Future strategies could integrate enforced expression of TFs that promote favourable states, such as KLF6 for T_RM_ differentiation or other TFs identified through systematic gain-of-function screenings^40–42,60^ with targeted depletion of TEX_term_ TFs. Such recipes can be refined with AI models. In summary, although our study focuses on CD8^+^ TEX_term_ and T_RM_ cell differentiation, the pipeline for identifying single-state TFs and ‘TF recipes’ can be adapted for other cell types, expanding cell therapy applications.

## Online content

Any methods, additional references, Nature Portfolio reporting summaries, source data, extended data, supplementary information, acknowledgements, peer review information; details of author contributions and competing interests; and statements of data and code availability are available at https://doi.org/10.1038/s41586-025-09989-7.

## Methods

### Dataset acquisition for mouse CD8^+^ T cell state multi-omics atlas

CD8^+^ T cell samples were collected from ten datasets, including those generated in this study (Extended Data Fig. 1e). In total, we analysed 121 experiments, comprising 52 ATAC-seq and 69 RNA-seq datasets, which were integrated to generate paired samples and served as input for the Taiji pipeline. The samples encompassed nine distinct CD8^+^ T cell subtypes: naive, TE, MP, T_RM_, T_EM_, T_CM_, TEX_prog_, TEX_eff_ and TEX_term_. Cell states were defined on the basis of established surface marker combinations and LCMV-specific tetramers, including IL7R, KLRG1, PD1, SLAMF6, CD101, Tim3, CD69, CD103, H2-Db LCMV GP33–41 and H2-Db LCMV GP276–286 or congenic markers for P14 (T cell receptor (TCR) specific for the LCMV GP33–41 peptide CD8^+^ T cells), in the context of either acute (LCMV–Armstrong) or chronic (LCMV–Clone 13) infection models. A complete summary of dataset sources, accession numbers, infection conditions and corresponding cell state definitions (sorting gates) is provided in Supplementary Table 1 and Extended Data Fig. 1e.

### TF regulatory networks construction and visualization

To perform integrative analysis of RNA-seq and ATAC-seq data, we developed Taiji v.2.0, which allows visualization of several downstream analysis–TF wave, TF–TF association and TF community analysis. Epitensor was used for the prediction of chromatin interactions. Putative TF binding motifs were curated from the latest CIS-BP database^61^. In this analysis, 695 TF genes were identified as having binding sites centred around ATAC-seq peak summits. The average number of nodes (genes) and edges (interactions) of the genetic regulatory networks across CD8^+^ T cell states were 15,845 and 1,325,694, respectively, including 695 (4.38%) TF nodes. On average, each TF regulated 1,907 genes, and each gene was regulated by 22 TFs.

### Identification of single-state and multi-state TF genes

We first identified universal TF genes with mean PageRank across nine cell states ranked as top 10% and coefficients of variation less than 0.5. In total, 54 universal TF genes were identified (Supplementary Table 1). The remaining 641 TF genes were candidates for single-state TF genes. To identify single-state TF genes, we divided the samples into two groups: target and background. The target group included all samples belonging to the cell state of interest, and the background group comprised the remaining samples. We then performed the normality test using Shapiro-Wilk’s method to determine whether the two groups were distributed normally, and we found that the PageRank scores of most (90%) samples followed a log-normal distribution. On the basis of the log-normality assumption, an unpaired *t*-test was used to calculate the *P* value. A *P* value cut-off of 0.05 and log_2_ fold change (log_2_FC) cut-off of 0.5 were used for calling lineage-specific TFs. In total, 255 specific TF genes were identified (Supplementary Table 2). Depending on whether the TF gene appeared in several cell states, they could be divided further into multi-state TF genes (Fig. 1e and Supplementary Table 2) and 136 single-state exclusive TF genes (Fig. 1d and Supplementary Table 2). Out of 255 single-state TF genes, 84 appear in TEX_term_ or T_RM_ cells. To identify the truly distinctive TF genes between TEX_term_ and T_RM_, we performed a second round of unpaired *t*-tests, between only TEX_term_ and T_RM_ cells (Supplementary Table 3). The same cut-offs, that is, *P* value of 0.05 and log_2_FC of 0.5, were applied to select TEX_term_ single-taskers and T_RM_ single-taskers. Out of 84 TF genes that did not pass the cut-off, 30 were identified as TEX_term_ and T_RM_ multi-taskers. The full workflow is summarized in Extended Data Fig. 2a.

### Identification of transcriptional waves

Combined with previous knowledge of the T cell differentiation path, TF waves are combinations of TFs that are particularly active in certain differentiation stages, revealing possible mechanisms of how TF activities are coordinated during differentiation. To be more specific, we clustered the TFs based on the normalized PageRank scores across samples. First, we performed principal component analysis (PCA) for dimensionality reduction of the TF score matrix. We retained the first ten principal components for further clustering analysis, which explained more than 70% of the variance (Extended Data Fig. 3b; left panel). We used the *k*-means algorithm for clustering analysis. To find the optimal number of clusters and similarity metric, we performed Silhouette analysis to evaluate the clustering quality using five distance metrics: Euclidean distance, Manhattan distance, Kendall correlation, Pearson correlation and Spearman correlation (Extended Data Fig. 3b; right panel). Pearson correlation was the most appropriate distance metric, as the average Silhouette width was highest of all five distance metrics. On the basis of these analyses, we identified seven distinct dynamic patterns of TF activity during immune cell development. We further performed functional enrichment analysis to identify gene ontology (GO) terms for these clusters.

### TF–TF collaboration network analysis and visualization

To build the TF–TF association networks, we first defined a set of relevant TFs for each context (TEX_term_ and T_RM_) by combining cell state-important and single-state TF genes, resulting in 159 TFs for TEX_term_ and 170 for T_RM_ cells. The analysis was based on a TF–regulatee network derived from Taiji, where we first consolidated sample networks by averaging the edge weights for each TF–regulatee pair. To reduce noise, regulatees with low variation across all TFs (s.d. ≤ 1) were removed. Subsequently, a TF–TF correlation matrix was generated by calculating the Spearman’s correlation of edge weights for each TF pair across their common regulatees. From this matrix, we constructed a graphical model using the R package ‘huge’^62^, which uses the Graphical Lasso algorithm and a shrunken empirical cumulative distribution function estimator. An edge between two TFs was established if their correlation was deemed significant by the model, controlled by a lasso penalty parameter (lambda) of 0.052. This value was chosen as it represents a local minimum on the sparsity lambda curve, resulting in approximately 15% of TF–TF pairs being connected. To validate this method, we estimated the false discovery rate by generating a null model through random shuffling of the TF–regulatee edge weights. Applying our algorithm to this null data identified zero interactions, confirming that our approach has a very low false discovery rate.

### TF community construction and visualization

Following construction of TF–TF association networks, we identified functionally related TF communities within each network. We applied the Leiden algorithm^63^, using modularity as the objective function and setting the resolution parameter to 0.9, as this value achieved the highest clustering modularity in our analysis. This procedure identified five distinct communities for each context (TEX_term_ and T_RM_). The final networks, with their detected communities, were visualized using the Fruchterman–Reingold layout algorithm^64^ to spatially represent the TF–TF association structure.

### Pathway enrichment analysis

The enriched functional terms in this study were analysed by the R package clusterProfiler v.4.0.5. We used the GO database, the Kyoto Encyclopedia of Genes and Genomes (KEGG) database and the Molecular Signatures Database for annotation. For GSEA, the genes were first ranked by the mean edge weight in corresponding samples and H, C5, C6 and C7 collections from the Molecular Signatures Database were used for annotation. A cut-off of *P* < 0.05 was used to select the significantly enriched GO terms and KEGG pathways.

### Heuristic score calculation through integration of the TF regulatory network and perturb-seq

We reasoned that (1) log_2_FC in expression due to TF KO and (2) TF–gene regulatory edge weights could be combined to provide heuristic scores for the regulatory effect of a TF on a target gene. For each guide RNA KO, Seurat’s FindMarkers() function was used to quantify log_2_FC in the expression of a gene with respect to the gScramble condition. Heuristic scores were calculated for each TF–gene pair by multiplying the gene log_2_FC with the corresponding edge weight from the Taiji analysis. Regulatees of a TF were annotated as high-confidence if the magnitude log_2_FC of the regulate exceeded 0.58 (corresponding to a fold change of 1.5 or its reciprocal) and if the edge weight belonged to the upper quantile of all edge weights attributed to the TF. The sign of the log_2_FC was used to determine whether the TF activated or repressed each target gene.

### Human TF activity and cell-state selectivity analysis

#### Taiji-based analysis of human multi-omic datasets.

TF activity was inferred using the Taiji pipeline applied to matched scRNA-seq and scATAC-seq datasets from various human cancers, including ccRCC (*n* = 2; PRJNA768891, GSE240822), GBM (GSE240822), BCC (GSE123814, EGAS00001006141), HNSCC (GSE139324, EGAS00001006141), HCC (GSE125449, EGAS00001006141) and RCC (PMID: 30093597; EGAS00001006141). Cell types were annotated using canonical marker gene expression and categorized into six main CD8^+^ T cell states: TEX, TEX_Prog_ (progenitor exhausted), T_Eff_ (effector), T_RM_, T_CM_/naive and proliferating. PageRank scores derived from Taiji were log-transformed, averaged within each T cell category, and then standardized using *z*-score normalization. Results were visualized with a focus on TF gene activity in T_RM_ and TEX populations.

#### TF gene expression comparison across human CD8^+^ T cell states.

To assess TF gene expression across diverse T cell states, raw count matrices from a published pan-cancer CD8^+^ T cell atlas (GSE156728) were reprocessed using Seurat’s standard workflow. The dataset encompassed T cells from 11 tumour types, including BC, BCL, CHOL, ESCA, FTC, MM, OV, PACA, RC, THCA and UCEC. Cell type annotations provided by the original study were retained and mapped to the following broad categories: T_RM_, TEX, T_EM_ (effector memory), T_M_ (memory), naive and T_K_ (cycling). Seurat’s AverageExpression function was used to compute average log_CPM_ expression for each TF gene in each T cell category, followed by *z*-score normalization. Data visualization emphasized comparisons between T_RM_ and TEX subsets.

### Mice and infections

C57BL/6/J, OT-1 (C57BL/6-Tg(TcraTcrb)1100Mjb/J), B6.Cg-*Rag2*^*tm1.1Cgn*^/J and CD45.1 (B6.SJL-*PtprcaPepcb*/BoyJ) mice were purchased from Jackson Laboratories. P14 mice have been described previously. Cas9 P14 mice were generated by crossing P14 mice with B6(C)-Gt(ROSA)26So rem1.1(CAG-cas9*,-EGFP)Rsky/J (Jackson Laboratories). Animals were housed in specific-pathogen-free facilities at the Salk Institute and University and at the University of North Carolina at Chapel Hill. All animal experiments were approved by the Institutional Animal Care and Use Committee. Mice were infected with 2 × 10^5^ plaque-forming units (PFU) LCMV–Armstrong by intraperitoneal injection or 2 × 10^6^ PFU LCMV Clone-13 by retro-orbital injection under anaesthesia.

### Viral titres

LCMV fluorescence focus unit titration was performed seeding Vero cells at a density of 30,000 cells per 100 μl in a 96-well flat-bottom plate in DMEM + 10% fetal bovine serum (FBS) + 2% HEPES + 1% penicillin-streptomycin. On the next day, tissues were homogenized on ice, spun down at 1,000*g* for 5 min at 4 °C and supernatants or serum were diluted in tenfold steps. Diluted samples were added to Vero cells and incubated at 37 °C, 5% CO_2_ for around 20 h. Subsequently, inocula were aspirated and wells were incubated with 4% paraformaldehyde for 30 min at room temperature before washing with PBS. VL-4 antibody (BioXCell) was conjugated using the Invitrogen AF488 conjugation kit and added to the wells in dilution buffer containing 3% BSA and 0.3% Triton (ThermoFisher Scientific) in PBS. Cells were incubated at 4 °C overnight before washing with PBS and counting foci under the microscope. The number of focus forming units was calculated using the formula: focus forming units per millilitre = number of plaques/(dilution× volume of diluted virus added to the plate).

### Cell isolation

Spleens were dissociated mechanically with 1-ml syringe plungers over a 70-μm nylon strainer. Spleens were incubated in ammonium chloride potassium buffer for 5 min. For isolation of small intestinal intraepithelial lymphocytes, Peyer’s patches were first removed by dissection. Intestines were cut longitudinally and then into 1-cm pieces and washed in PBS. Pieces were incubated in 30 ml HBSS with 10% FBS, 10 mM HEPES and 1 mM dithioerythritol with vigorous shaking at 37 °C for 30 min. Supernatants were collected, washed and isolated further using 40%/67% discontinuous Percoll density centrifugation for 20 min at room temperature with no brake.

### Cell lines and in vitro cultures

B16-GP33 melanoma cell lines were cultured in DMEM (Invitrogen) with 10% FBS, 1% penicillin-streptomycin and 250 μg ml^−1^ G418 (Invitrogen, catalogue no. 10131027). The MCA-205 tumour line (Sigma) was maintained in RPMI supplemented with 10% FBS, 300 mg l^−1^ l-glutamine, 100 U ml^−1^ penicillin, 100 mM sodium pyruvate, 100 μM non-essential amino acids, 1 mM HEPES, 55 μM 2-mercaptoethanol and 0.2% plasmocin mycoplasma prophylactic (InvivoGen). All the tumour cell lines were used for experiments when in the exponential growth phase. For in vitro T cell culture, splenocytes were activated in RPMI 1640 medium (Invitrogen) containing 10% FBS and 1% penicillin-streptomycin, 2 mM l-glutamine, 0.1 mg ml^−1^ GP33, beta-mercaptoethanol 50 mM and 10 U ml^−1^ IL-2.

### Tumour engraftment and treatment of tumour-bearing mice

A total of 3 × 10^5^ B16-GP33 (Fig. 5a), 5 × 10^5^ B16-GP33 tumour cells (Fig. 5n,o) were injected subcutaneously in 100 μl PBS. Around 0.5–1 × 10^6^ Cas9^+^ P14 T cells with CD45.1 markers were transferred to tumour on day 7 without pre-radiation of tumour-bearing mice. Tumours were measured every 2–3 days post-tumour engraftment for indicated treatments and sizes calculated. Tumour volume was calculated as volume = (length × width^2^)/2. For antibody-based treatment, tumour-bearing mice were treated with anti-PD1 antibody (200 μg per injection, clone RMP1–14, BioXcell) twice per week from day 7 post-tumour implantation. Tumour growth was measured twice per week with calipers. Survival events were recorded each time a mouse reached the endpoint (tumour volume greater than or equal to 1,500 mm^3^). Tumour weights were measured on day 23 for Fig. 5a and on day 25 for Fig. 5m–o. All experiments were conducted according to the Salk Institute Animal Care and Use Committee and the University of North Carolina at Chapel Hill Animal Care and Use Committee.

### Tumour digestion and cell isolation

For the data shown in Fig. 5, tumours were minced into small pieces in RPMI containing 2% FBS, DNase I (0.5 μg ml^−1^; Sigma-Aldrich), and collagenase (0.5 mg ml^−1^; Sigma-Aldrich) and kept for digestion for 30 min at 37 °C with 70-μm cell strainers (VWR). Filtered cells were incubated with ammonium chloride potassium lysis buffer (Invitrogen) to lyse red blood cells, mixed with excess RPMI 1640 medium (Invitrogen) containing 10% FBS and 1% penicillin-streptomycin, and centrifuged at 400*g* for 5 min to obtain a single-cell suspension.

### Proteasome activity analysis

For experiments involving the Proteasome Activity Probe (R&D systems), cells of interest were incubated with the probe at concentration of 2.5 mM for 2 h at 37 °C in PBS. Samples were washed and then stained with Zombie NIR viability dye (Biolegend) in PBS at 4 °C for 15 min. Samples were then stained with some variation of the following antibodies for 30 min in fluorescence-activated cell sorting (FACS) buffer on ice: CD45-BV510 (BD Biosciences), CD45.2-BV510 (Biolegend), CD45.1-PE-Cy7 (Invitrogen), CD4-APC Fire 810 (Biolegend), CD11b-Alexa Fluor 532 (Invitrogen), CD8-Spark NIR 685 (Biolegend), CD44-Brilliant Violet 785 (Biolegend), CD62L-BV421 (BD Biosciences), PD1-BB700 (BD Biosciences), TIM3-BV711 (Biolegend), LAG3-APC-eFluor 780 (Invitrogen), SlamF6-APC (Invitrogen), CD39-Superbright 436 (Invitrogen), CX3CR1-PE/Fire 640 (Biolegend), CD69-PE-Cy5 (Biolegend), GITR-BV650 (BD Biosciences) and CD27-BV750 (BD Biosciences). Samples were collected on a Cytek Northern Lights and analysed using Cytek SpectraFlo software.

### Tumour experiment for proteasome assay

For the data shown in Fig. 2, MCA-205 fibrosarcomas (2.5 × 10^5^) were established by subcutaneous injection into the right flank of C57BL/6 mice. After 12–14 days of tumour growth, spleens, draining lymph nodes and tumours from groups of mice were collected and tumours were processed using the Mouse Tumor Dissociation Kit and gentleMACS dissociator (Miltenyi Biotec) according to the manufacturer’s protocol. For purification experiments, samples were pre-enriched using the EasySep Mouse CD8^+^ T Cell Isolation Kit (Stemcell Technologies) according to the manufacturer’s protocol, stained with Live-or-Dye PE Fixable Viability Stain (Biotium) and CD8a-APC (Invitrogen) and live CD8^+^ cells were sorted using the FACSAria II cell sorter. CD8^+^ spleen and pooled TIL samples were washed in PBS and frozen for RNA-seq analysis. For adoptive cellular therapy experiments, B16-GP33 melanomas were established subcutaneously by injecting 5.0 × 10^5^ cells into the right flank of CD45.1 mice and tumour-bearing hosts were irradiated with 5 Gy 24 h before T cell transfer. By contrast, mice used in the experiments shown in Fig. 5 were not irradiated before T cell transfer. After 7 days of tumour growth, 1.5 × 10^6^ CD45.2 OT-1 T cells and 1.5 × 10^6^ CD45.1/CD45.2 P14 T cells were infused in 100 μl PBS into the tail vein in tumour-bearing mice. Tumours were collected 14 days after adoptive cell transfer and CD8 TILs were analysed for proteasome activity. All experiments were conducted in accordance with the guidelines of the University of North Carolina at Chapel Hill Animal Care and Use Committee.

### Proteasome^high^/proteasome^low^ T cell adoptive transfer experiment

For the adoptive transfer experiment involving proteasome^high^ and proteasome^low^ tumour-specific OT-1 T cells (Fig. 2l), whole splenocytes from OT-1 mice were activated with 1 μg ml^−1^ OVA_257–264 peptide and expanded for 7 days in the presence of 200 U ml^−1^ rhIL-2 (NCI). On day 7, OT-1 cells were FACS-sorted based on proteasome activity to isolate proteasome^high^ and proteasome^low^ OT-1 populations. A total of 2.5 × 10^5^ sorted OT-1 cells were injected into C57BL/6 mice bearing B16F1-OVA melanomas. Tumours were established by subcutaneous injection of 3 × 10^5^ B16F1-OVA cells into the right flank 7 days before T cell transfer. Recipient mice were preconditioned with 5 Gy total body irradiation 24 h before adoptive transfer. Tumour growth was measured every other day with calipers.

For Extended Data Fig. 5c, MCA-205 fibrosarcomas (2.5 × 10^5^) were established by means of subcutaneous injection into the right flank of C57BL/6 mice. After 14 days of tumour growth, live CD45^+^CD8^+^CD44^+^PD1^+^ T cells were sorted from tumours on the basis of proteasome activity (high versus low) using the FACSAria II cell sorter. A total of 2.5 × 10^4^ cells were then injected into the 2-day MCA-205-bearing *RAG2*^−/−^ hosts (*n* = 5 per group) and tumour growth was monitored every other day starting on day 4. All experiments were conducted in accordance with the guidelines of the University of North Carolina at Chapel Hill Animal Care and Use Committee.

### Retrovirus transduction and adoptive transfer

For overexpression of the gRNA retrovirus vector, 293T cells were transfected with the Eco-helper and MSCV gRNA vectors. At 48 h and 72 h later, supernatant containing retroviral particles was ready for transduction. Donor P14 splenocytes were activated in vitro by 0.1 mg ml^−1^ GP33 and 10 U ml^−1^ IL-2 at 37 °C for 24 h, then spin-transduced (1,500*g*) with fresh retrovirus supernatant from 293T cells for 90 min at 30 °C in the presence of 5 μg ml^−1^ polybrene.

### CRISPR–Cas9/RNP nucleofection

Naive CD8^+^ T cells were enriched from spleen using the EasySep Mouse CD8^+^ T cell Isolation Kit (Stemcell Technologies). sgRNAs targeting *Zscan20*, *Jdp2*, *Etv5*, *Prdm1* and *Hic1* genes or the mouse or human genome non-targeting scramble (control) were obtained from Synthego, Integrated DNA technologies (IDT) and GeneScript (Supplementary Table 5). Cas9 RNP was prepared immediately before experiments by incubating 1 μl sgRNA (stock, 3 nmol in 10 μl water), 0.6 μl Cas9 nuclease (IDT; stock, 62 μM) and 3.4 μl RNase-free water at room temperature for 10 min. Nucleofection of naive CD8^+^ T cells was performed using a Lonza P3 primary cell kit and program DN100 with 4D-Nucleofector (Lonza Bioscience) for mouse and EO115 for human stimulated T cells. Each nucleofection reaction consisted of approximately 5–10 × 10^6^ cells in 20 μl of nucleofection reagent and mixed with 5 μl of RNP:Cas9 complex. After electroporation, 100 μl of T cell culture medium was added to the well to transfer the cells to 1.5 ml Eppendorf tubes. The cells were rested at 37 °C for 3 min. For in vivo adoptive transfer, cells were resuspended in PBS at the desired concentration and transferred adoptively into recipient mice.

### CRISPR gene editing validation by Sanger sequencing

Genomic DNA was isolated from both KO-induced CD8^+^ T cells and control cells using a Quick-DNA MicroPrep Kit (Zymo). Genomic DNA concentrations were quantified using a NanoDrop One spectrophotometer (ThermoFisher Scientific). Following isolation, PCR amplification was performed with 2× Phusion Plus Green PCR Master Mix (ThermoFisher Scientific) and the respective validation primers under the following conditions: 98 °C for 5 min; 35× 98 °C for 10 s, 69 °C for 20 s, 72 °C for 20–30 s kb^−1^; 72 °C for 2 min; hold at 10 °C). The PCR products were resolved on a 2% agarose gel with SYBR Safe DNA Gel Stain (Invitrogen), and the appropriate bands on the gel were extracted and purified with a Gel DNA Recovery Kit (Zymo). Concentrations of purified amplicon samples were measured and then sent for sequencing with primers designed using Benchling’s Primer3 tool. The samples with KOs were compared with wild-type controls using EditCo’s Ice Analysis software, providing the indel percentages, KO score and the indel distributions used to assess editing efficiency. Indel percent ranged from 56% to 97%, and the KO score throughout experiments ranged from 32 to 74.

### Flow cytometry, cell sorting and antibodies

Both single-cell suspensions were incubated with Fc receptor-blocking anti-CD16/32 (BioLegend) on ice for 10 min before staining. Cell suspensions were first stained with Red Dead Cell Stain Kit (ThermoFisher) for 10 min on ice. Surface proteins were then stained in FACS buffer (PBS containing 2% FBS and 0.1% sodium azide) for 30 min at 4 °C. To detect cytokine production ex vivo, cell suspensions were resuspended in RPMI 1640 containing 10% FBS, stimulated by 50 ng ml^−1^ phorbol 12-myristate 13-acetate and 3 μM ionomycin in the presence 2.5 μg ml^−1^ Brefeldin A (BioLegend, catalogue no. 420601) for 4 h at 37 °C. Cells were processed for surface marker staining as described above. For intracellular cytokine staining, cells were fixed in BD Cytofix/Cytoperm (BD, catalogue no. 554714) for 30 min at 4 °C, then washed with 1× permeabilization buffer (Invitrogen, catalogue no. 00–8333-56). For transcription factor staining, cells were fixed in FOXP3/transcription factor fixation/permeabilization buffer (Invitrogen, catalogue no. 00–5521-00) for 30 min at 4 °C, then washed with 1× permeabilization buffer. Cells were then stained with intracellular antibodies for 30 min at 4 °C. Samples were processed on an LSR-II flow cytometer (BD Biosciences) and data were analysed with FlowJo v.10 (TreeStar). Cells were sorted either on a FACSAria III sorter or a Fusion sorter (BD Biosciences). The following antibodies (clone nos.) against mouse proteins were used: anti-CD8a (53–6.7), anti-PD1 (29F.1A12), anti-CX3CR1 (SA011F11), anti-SLAMF6 (13G3), anti-CD38 (90), anti-CD39 (24DMS1), anti-CD101 (Moushi101), anti-KRLG1 (2F1), anti-CD69 (H1.2F3), anti-CD103 (M290), anti-CD62L (MEL-14), anti-TIM3 (RMT3–23), anti-Ly5.1 (A20), anti-Ly5.2 (104), anti-IFNγ (XMG1.2) and anti-TNF (MP6-XT22). The following antibodies (clone nos.) against human proteins were used: anti-CD8a (RPA-T8), anti-CD4 (SK3), anti-CD45RA (H100), anti-CD45RO (UCHL1), anti-CCR7 (G043H7), anti-CD62L (DREG-56), anti-CD69 (FN50), anti-CD103 (Ber-ACT8), anti-CXCR6 (K041E5), anti-PD1 (EH12.2H7), anti-CD38 (HIT2), anti-CD39 (A1), anti-LAG3 (11C3C65), anti-TIM3 (F38–2E2), anti-TIGIT (A15153G), anti-IFNγ (4S.B3), anti-TNF (MAb11), anti-IL-2 ( JES6–5H4), anti-GZMB (QA16A02) and anti-G4S Linker (E7O2V). Antibodies were purchased from Invitrogen, Biolegend, Cell Signaling or eBiosciences.

### In vivo individual TF KO phenotyping

To assess the functional impact of individual TF gene KOs in CD8^+^ T cells, we used Cas9-expressing P14 donor cells (LCMV-specific TCR transgenic mice, CD45.1 congenic) transduced with green fluorescent protein (GFP)-expressing retroviral vectors encoding individual gRNAs. Transductions were performed on the day of adoptive transfer without previous sorting. Without sorting, transduced donor cells (0.5–1 × 10^5^) were transferred immediately into congenically distinct Cas9-expressing wild-type recipient mice (CD45.2) infected 1 day previously with either LCMV–Clone 13 or LCMV–Armstrong strains. At least day 20 post-infection, spleens from the Clone 13 model and spleens and small intestines from the Armstrong model were collected. Single-cell suspensions were prepared and analysed by flow cytometry. Live, single cells were first gated on CD8^+^ cells, followed by gating on CD45.1^+^ P14 donor CD8^+^ T cells. Successfully transduced (gRNA^+^) cells were identified by GFP expression, which ranged from 10% to 70% of P14 CD8^+^ T cells across experiments. Because of variability in the number of GFP^+^ donor P14 CD8^+^ T cells obtained from different experiments, all phenotypic analyses were performed in the GFP^+^(gRNA^+^) CD45.1^+^CD8^+^ population. PD1 positive and negative cells, exhaustion subsets (TEX_term_:PD1^+^SLAMF6^−^CX3CR1^−^ and TEX_prog_:PD1^+^SLAMF6^+^C X3CR1^−^, TEX_eff_:PD1^+^CX3CR1^+^) or expression of phenotypic markers was reported as a percentage within the gRNA^+^(GFP^+^) P14 CD8^+^ T cell population to ensure consistency across samples.

### Co-transfers of control and TF gene KO/overexpression P14 CD8^+^ T cells in infection or tumour models

Naive CD8^+^ T cells were isolated from the spleens and lymph nodes of Cas9-expressing LCMV TCR transgenic (Cas9 P14) or P14 mice using an EasySep Mouse CD8^+^ T Cell Isolation Kit (STEMCELL Technologies). Purified P14 cells were activated for about 24 h on plates coated with goat anti-hamster IgG (ThermoFisher), followed by 1 μg ml^−1^ hamster anti-mouse CD3 and 1 μg ml^−1^ hamster anti-mouse CD28 antibodies (ThermoFisher), in complete T cell medium (RPMI 1640 supplemented with 10% FBS (HyClone), 55 μM 2-mercaptoethanol, 100 IU ml^−1^ penicillin-streptomycin and 1% HEPES). After activation, cells were transduced with retroviruses encoding *Klf6* overexpression or gRNAs targeting *Hic1* or *Zscan20* and cultured with 20 IU ml^−1^ IL-2, 2.5 ng ml^−1^ IL-7 and 2.5 ng ml^−1^ IL-15 (PeproTech). At 48 h post-transduction, reporter expression was confirmed by flow cytometry. Donor cell mixes were prepared using control versus *Klf6*-overexpressing cells (Fig. 4) or gScramble versus g*Hic1*/g*Zscan20* cells (Fig. 5). For LCMV infection studies, 1.5 × 10^5^ transduced P14 CD8^+^ T cells were transferred into recipient mice, followed by infection with either 2 × 10^5^ PFU LCMV–Armstrong (acute infection, intraperitoneal) or 2 × 10^6^ PFU LCMV–Clone-13 (persistent infection, intravenous). For tumour studies, 5 × 10^5^ to 1 × 10^6^ transduced T cells (gScramble versus gTF) were transferred on day 7 after B16-GP33 tumour implantation. All experiments were conducted according to guidelines of the University of North Carolina at Chapel Hill Animal Care and Use Committee.

### Perturb-seq screening using the retroviral transcriptional factor library

#### Dual-guide direct-capture retroviral sgRNA vector.

To generate a dual-guide sgRNA vector (MSCV-hU6-mU6-SV40-EGFP), we replaced the hU6 RNA scaffold region of the previously described retroviral sgRNA vector MG-guide^65^ with an additional scaffold^66^ and the mouse U6 promoter.

#### Dual-guide direct-capture retroviral library construction.

For the curated gene list containing 21 TF genes, a total of four gRNA sequences distributed on two individual constructs were designed for each gene. To construct the library, a customized double-strand DNA fragment pool containing 80 oligonucleotides targeting those 19 TF genes and four scramble gRNAs (each oligonucleotide contains two guides targeting the same gene) (Supplementary Table 5) was ordered from IDT. The dual-guide library was generated using an In-Fusion (Takara) reaction. In brief, the gRNA containing DNA fragment pool was combined in MG-guide vector linearized with *Bpi*I (ThermoFisher). The construct was then transformed into Stellar competent cells (Takara) and amplified, and the resulting intermediate, individual, construct was assessed for quality using Sanger sequencing. Individual dual-gRNA vectors were then combined. For quality control, sgRNA skewing was measured using the MAGeCKFlute^67^ to monitor how closely sgRNAs are represented in a library.

#### In vivo screening.

Retrovirus was generated by co-transfecting HEK293 cells with the dual-guide, direct-capture retroviral TF library and the packaging plasmid pCL-Eco. Supernatants were collected at 48 h and 72 h post-transfection then stored at −80 °C. Cas9-expressing P14 CD8^+^ T cells were transduced with the viral supernatant to achieve a transduction efficiency of 20–30%. To ensure sufficient representation of control cells in downstream analysis, 50% of the viral mixture consisted of retrovirus encoding a non-targeting control gRNA vector. For in vivo experiments, 5 × 10^4^ transduced P14 cells were transferred intravenously into Cas9-expressing, puromycin-resistant C57BL/6 recipient mice infected 1 day previously with either LCMV–Clone-13 or LCMV–Armstrong strain. A total of 25 LCMV–Clone-13-infected mice were used for five biological replicates and ten LCMV–Armstrong-infected mice were used for three biological replicates. Each biological replicate was labelled using hashtag antibodies (BioLegend, TotalSeq-C) to enable sample demultiplexing and statistical analysis. At least 18 days post-infection, donor-derived P14 CD8^+^ T cells were sorted and pooled for Perturb-seq analysis. Preliminary tests indicated that T cells expressing gRNA in vivo exhibit a greater tendency for gRNA silencing over extended periods compared with ex vivo cultured cells, despite initial successful KOs. To mitigate gRNA barcode silencing, we collected tissue between days 18 and 23. Sorted EGFP^+^ P14 CD8^+^ T cells were resuspended and diluted in 10% FBS RPMI at a concentration of 1 × 10^6^ cells ml^−1^. Both the gene expression library and the CRISPR screening library were prepared using a Chromium Next GEM Single Cell 5′ kit with Feature Barcode technology for CRISPR Screening (10x Genomics). In brief, the single-cell suspensions were loaded onto the Chromium Controller according to their respective cell counts to generate 10,000 single-cell gel beads in emulsion per sample. Each sample was loaded into four separate channels. Chromium Next GEM Single Cell 5′ Kit v.2 (catalogue no. 1000263), Chromium 5′ Feature Barcode Kit (catalogue no. 1000541), 5′ CRISPR Kit (catalogue no. 1000451), Chromium Next GEM Chip K Single Cell Kit (catalogue no. 1000287), Dual Index Kit TT Set A (catalogue no. 1000215), Dual Index Kit TN Set A (catalogue no. 1000250) (10x Genomics) in total were used for each reaction. The resulting libraries were quantified and quality checked using TapeStation (Agilent). Samples were diluted and loaded onto a NovaSeq (Illumina) using a 100 cycle kit to obtain a minimum of 20,000 paired-end reads (26 × 91 bp) per cell for the gene expression library and 5,000 paired-end reads per cell for the CRISPR screening library, yielding an average of 42,639; 36,739 and 53,413 reads aligned from cells from in vivo LCMV–Clone-13, in vivo LCMV–Armstrong infection and in vitro donor respectively.

### Data analysis

Alignments and count aggregation of gene expression and sgRNA reads were completed using Cell Ranger (v.7.0.1). Gene expression and sgRNA reads were aligned using the Cell Ranger multi count command with default settings. Gene expression reads were aligned to the mouse genome (mm10 from ENSEMBL GRCm38 loaded from 10x Genomics). The median average of four, two and 33 unique molecular identifiers (UMIs) were detected from cells from in vivo LCMV–Clone 13 and LCMV–Armstrong infection, and in vitro donor, respectively. Droplets with sgRNA UMI passing of default Cell Ranger CRISPR analysis Protospacer UMI threshold were used in further analysis. The filtered feature matrices were imported into Seurat^68^ (v.4.3.0) to create assays for a Seurat object containing both gene expression and CRISPR guide capture matrices. Cells detected with sgRNAs targeting two or more genes were then removed to avoid interference from multi-sgRNA-transduced cells. Low-quality cells with fewer than 200 detected genes, more than 10% mitochondrial reads and less than 5% ribosomal reads were discarded. A total of 17,257 cells (Clone-13) and 15,211 cells (Armstrong) were passed through quality filtering and were used for downstream analysis. Count data were normalized by a global-scaling normalization method and linear transformed^69^. Cluster-specific genes were identified using the FindAllMarkers function of Seurat. We used Nebulosa^70^ to recover signals from sparse features in single-cell data and made gRNA density plots with scCustomize^71^ based on kernel density estimation. In each biological replicate (Clone-13, *n* = 5; Armstrong, *n* = 3), the percentage cluster distribution of cells with each TF gRNA vector was calculated. Among two gRNA vectors per target TF, the gRNA vector with higher TEX_term_ reduction was shown in Fig. 3d and used for Perturb-seq in LCMV–Armstrong infection (Supplementary Table 5). Two-way ANOVA with Fisher’s LSD test was performed to determine statistical significance. Differentially expressed genes were identified using the MAST model^72^; the results were then used as inputs for GSEA to evaluate the effect on selected pathways. Genes with *P* value < 0.05 were considered as differentially expressed genes.

UMAP plots were generated by calculating UMAP embeddings using Seurat and then plotting them as scatter plots using ggplot2. Kernel density calculations for each gRNA were performed on UMAP embeddings using the MASS package using the kde2d function. The kernel density results were plotted as a raster layer with ggplot2 over the UMAP scatter plots. Finally, density contour lines were added using ggplot2’s built-in two-dimensional kernel density contour geom (geom_density_2d).

### ATAC-seq library preparation and sequencing

ATAC-seq was performed as described previously^73^. In brief, 5,000–50,000 viable cells were washed with cold PBS, collected by centrifugation, then lysed in resuspension buffer (RSB) (10 mM Tris-HCl, pH 7.4, 10 mM NaCl, 3 mM MgCl_2_) supplemented with 0.1% NP40, 0.1% Tween-20 and 0.01% digitonin. Samples were incubated on ice for 3 min, then washed out with 1 ml RSB containing 0.1% Tween-20. Nuclei were pelleted by centrifugation at 500*g* for 10 min at 4 °C then resuspended in 50 μl transposition mix (25 μl 2× TD buffer, 2.5 μl transposase (100 nM final), 16.5 μl PBS, 0.5 μl 1% digitonin, 0.5 μl 10% Tween-20, 5 μl H_2_O) and incubated at 37 °C for 30 min in a thermomixer with 1,000 rpm mixing. DNA was purified using a Qiagen MinElute PCR cleanup kit, then amplified by PCR using indexed oligos. The optimal number of amplification cycles for each sample was determined by quantitative PCR. Libraries were size-selected using AmpureXP beads and sequenced using an Illumina NextSeq500 for 75-bp paired-end reads.

### ATAC-seq analysis

Paired-end 42-bp or paired-end 75-bp reads were aligned to the *Mus musculus* mm10 genome using Burrow–Wheeler aligner^74,75^ with parameters ‘bwa mem -M -k 32’. ATAC-seq peaks were called using the MACS2 (ref. 76) program using parameters ‘callpeaks -qvalue 5.0e-2 – shift −100 –extsize 200’. Differentially accessible regions were identified using DESeq2 (ref. 77). Batch effect was removed using limma^78^. Heatmap visualization of ATAC-seq data was performed using pheatmap.

### scRNA-seq metadata analysis

Analysis was performed primarily in R (v.3.6.1) using the package Seurat^68,79^ (v.3.1), with the package tidyverse^80^ (v.1.2.1) used to organize data and the package ggplot2 (v.3.2.1) to generate figures. scRNA-seq data from GSE10898, GSE99254, GSE98638, GSE199565 and GSE181785 were filtered to keep cells with a low percentage of mitochondrial genes in the transcriptome (less than 5%) and between 200 and 3,000 unique genes to exclude poor quality reads and doublets. Cell cycle scores were regressed when scaling gene expression values and TCR genes were regressed during the clustering process, which was performed with the Louvain algorithm within Seurat and visualized with UMAP. To quantify the gene expression patterns, we used Seurat’s module score feature to score each cluster based on its per cell expression of TFs.

To obtain Extended Data Fig. 5a, raw single-cell count data and cell annotation data were downloaded from NCBI GEO^44^ (GSE99254). Count data were normalized and transformed by derivation of the residuals from a regularized negative binomial regression model for each gene (SCT normalization method in Seurat^68^, v.4.1.1), with 5,000 variable features retained for downstream dimensionality reduction techniques. Integration of data was performed on the patient level with Canonical Correlation Analysis as the dimension reduction technique^81^. PCA and UMAP dimension reduction were performed, with the first 50 principal components used in UMAP generation. Cells were clustered using the Louvain algorithm with multi-level refinement. The data was subset to CD8^+^ T cells, which were identified using the labels provided by Guo et al.^65^. Cell type labels were confirmed by (1) SingleR^82^ (v.1.8.1) annotation using the ImmGen^83^ database obtained through celda (v.1.10), (2) cluster marker identification and (3) cell type annotation with the ProjecTILs T cell atlas^7^ (v.2.2.1). After sub-setting to CD8^+^ T cells, cells were again normalized using SCT normalization, with 3,000 variable features retained for dimension reduction. Owing to the low number of cells on the per-patient level, HArmstrongony^84^ (v.1.0) rather than Seurat was used to integrate data at the patient level. PCA and UMAP dimensionality reduction were performed as above.

### Statistical analyses

Statistical tests for flow cytometry data were performed using Graphpad Prism v.10. *P* values were calculated using either two-tailed unpaired Student’s *t*-tests, one-way ANOVA or two-way ANOVA as indicated in each figure. Linear regressions were performed using the ordinary least squares method in R (v.3.6.1). All data were presented as the mean ± s.e.m. *P* values were represented as follows: ****P < 0.0001, ***P < 0.001, **P < 0.01 and *P < 0.05.

## Extended Data

**Extended Data Fig. 6 |:**
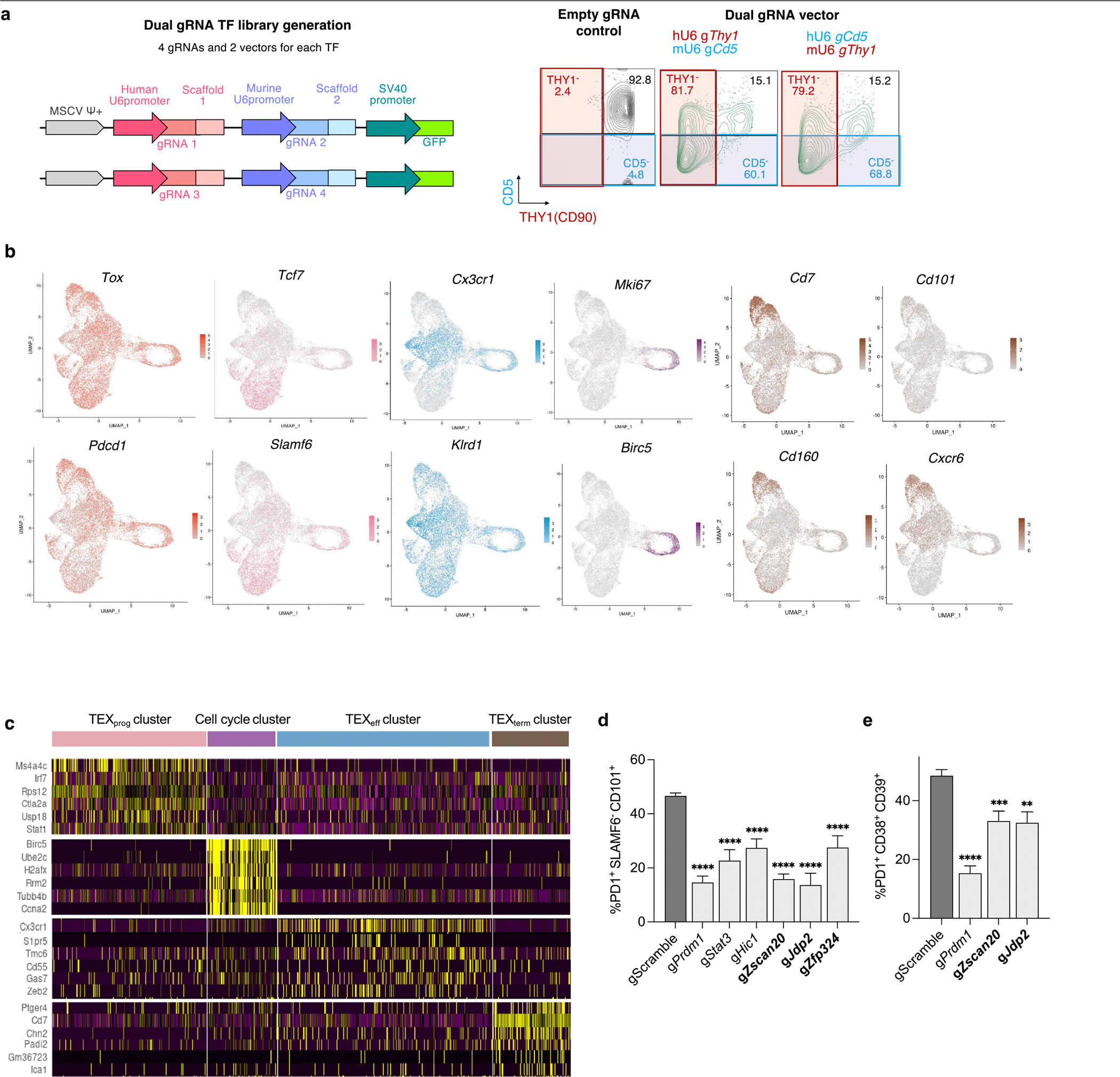
In vivo Perturb-seq with dual-guide RNA in LCMV chronic infection and individual validation. **a**, Retroviral vector design for dual-gRNA delivery. **b**, Feature plot of differentiation markers (Pan-exhaustion: red, TEX_prog_: pink, TEX_eff_: blue, Cell cycle: purple, TEX_term_: brown). **c**, Heatmap of marker gene expression across cell state clusters identified by Seurat’s Find Markers() function. **d**, Frequency of PD1^+^SLAMF6^−^CD101^+^ cells (**d**). Frequency of CD38^+^CD39^+^ double-positive cells (**e**). Statistical analysis: Ordinary one-way ANOVA with Dunnett’s multiple comparisons test versus gScramble (n ≥ 5 from ≥2 biological replicates). Data are presented as mean ± s.e.m. ****P < 0.0001; ***P < 0.001; **P < 0.01; *P < 0.05.

**Extended Data Fig. 7 |:**
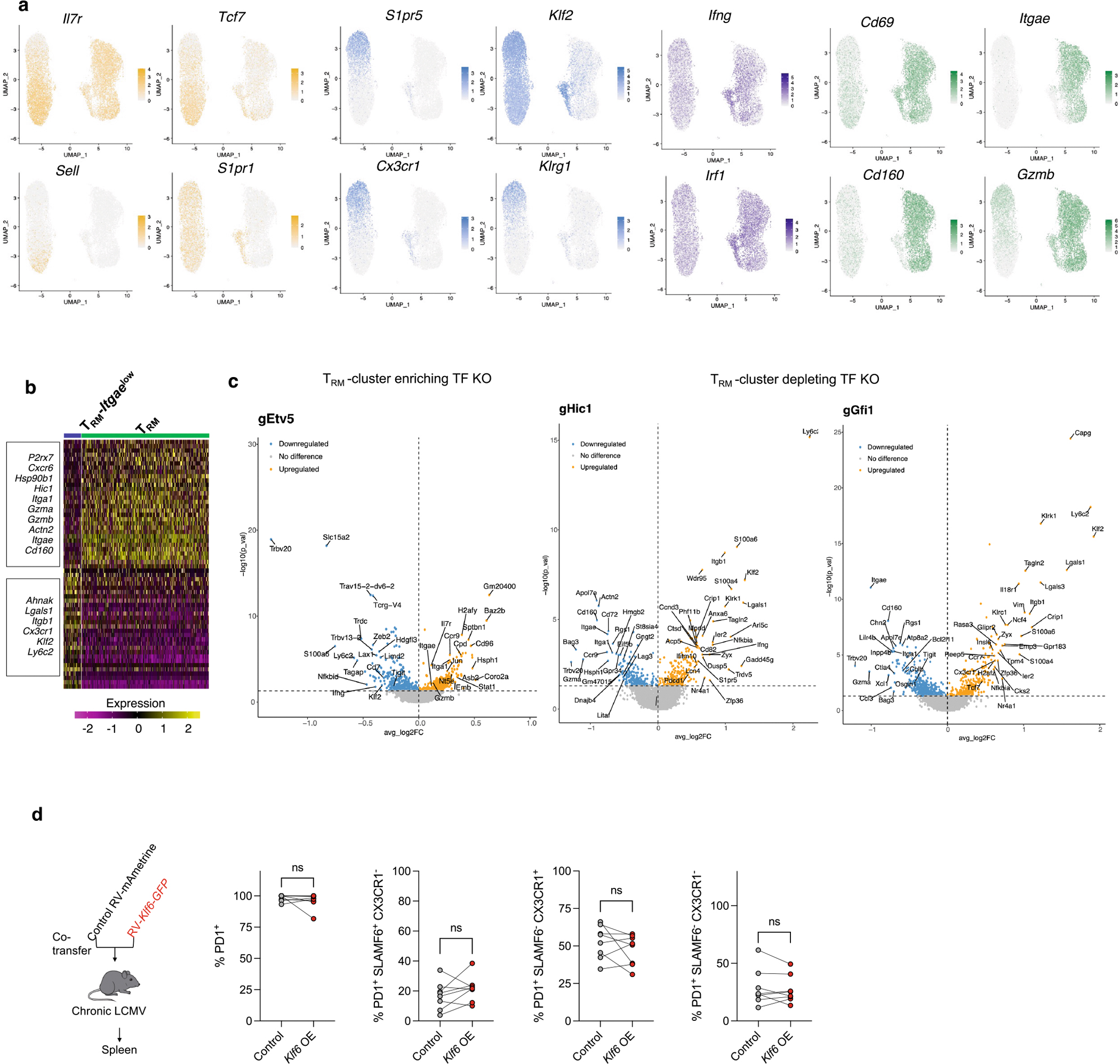
Single-cell transcriptomic profiling of CD8^+^ T cells from Perturb-seq in acute LCMV infection and validation of the selectivity of T_RM_ single-state TF, *Klf6* overexpression. **a**, Feature plots of differentiation marker genes (T_CM_: yellow, T_EM_: blue, T_RM_-*Itgae*^low^: dark blue, T_RM_: green). **b**, Heatmap of differentially expressed genes between T_RM_-*Itgae*^low^ and T_RM_ clusters. **c**, Volcano plots of differentially expressed genes in Cas9^+^ P14 CD8^+^ T cells expressing *gEtv5*, *gArid3a*, g*Hic1*, or *gGfi1*. **d**, T_RM_ single-state TF, *Klf6* overexpression does not accelerate T cell exhaustion. Experimental setup: *Klf6-*RV or control-RV transduced P14 CD8^+^ T cells co-transferred into mice infected with chronic LCMV-Clone-13, Quantification of the frequency of PD1^+^, TEX_prog_ (PD1^+^SLAMF6^+^CX3CR1^−^), TEX_eff_ (PD1^+^SLAMF6^−^CX3CR1^+^) and TEX_term_ (PD1^+^SLAMF6^−^CX3CR1^−^) populations. Paired t-tests (n ≥ 6 from ≥2 biological replicates). Data are presented as mean ± s.e.m. ****P < 0.0001, ***P < 0.001, **P < 0.01, *P < 0.05.

**Extended Data Fig. 8 |:**
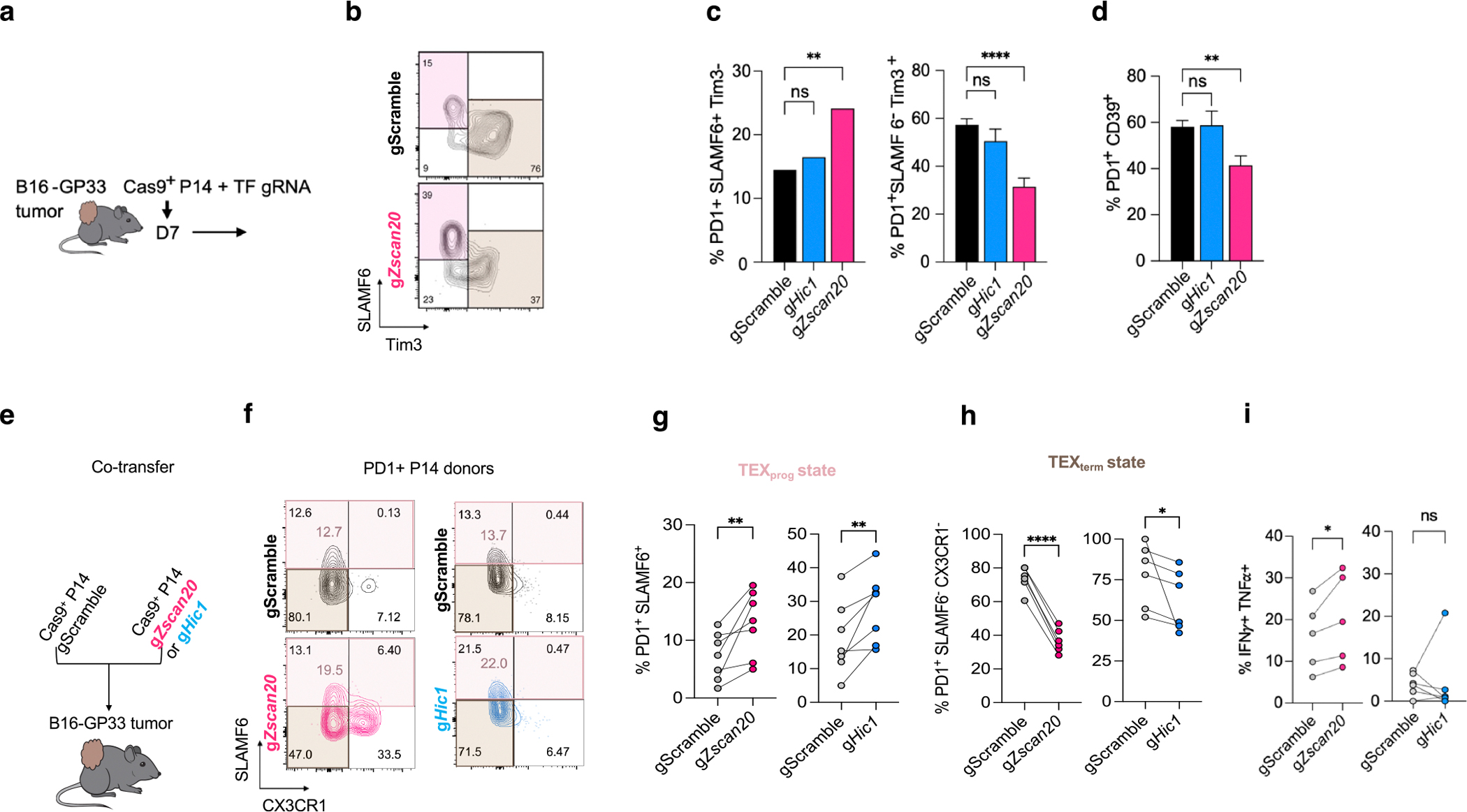
Depletion of TEX_term_-single state TF, *Zscan20* and TEX_term_ and T_RM_ multi-state TF, *Hic1* reduces T cell exhaustion. **a**, Experiment design. **b**, Representative flow plots of PD1^+^ P14 cells stained for SLAMF6 and TIM3. **c**, quantification of TEX_prog_ (PD1^+^SLAMF6^+^TIM3^−^) and exhausted (PD1^+^SLAMF6^−^TIM3^+^) subsets. **d**, Frequency of PD1^+^CD39^+^ double-positive cells. **e**, Co-transfer of Cas9^+^ P14 CD8^+^ T cells transduced with RV-*gZscan20* or RV-*gHic1*, mixed with gRNA control RV transduced cells into B16-GP33 tumor-bearing mice. **f**, Representative flow plots of PD1^+^ P14 CD8^+^ T cells stained for SLAMF6 and CX3CR1. **g-i**, Quantification of (**g**) TEX_prog_ (PD1^+^SLAMF6^+^TIM3^−^), (**h**) TEX_term_ (PD1^+^SLAMF6^−^TIM3^+^), and (**i**) IFN*γ*^+^TNF^+^ population. Paired t-tests (n ≥ 6 from ≥2 biological replicates). Data are presented as mean ± s.e.m. ****P < 0.0001, ***P < 0.001, **P < 0.01, *P < 0.05.

**Extended Data Fig. 9 |:**
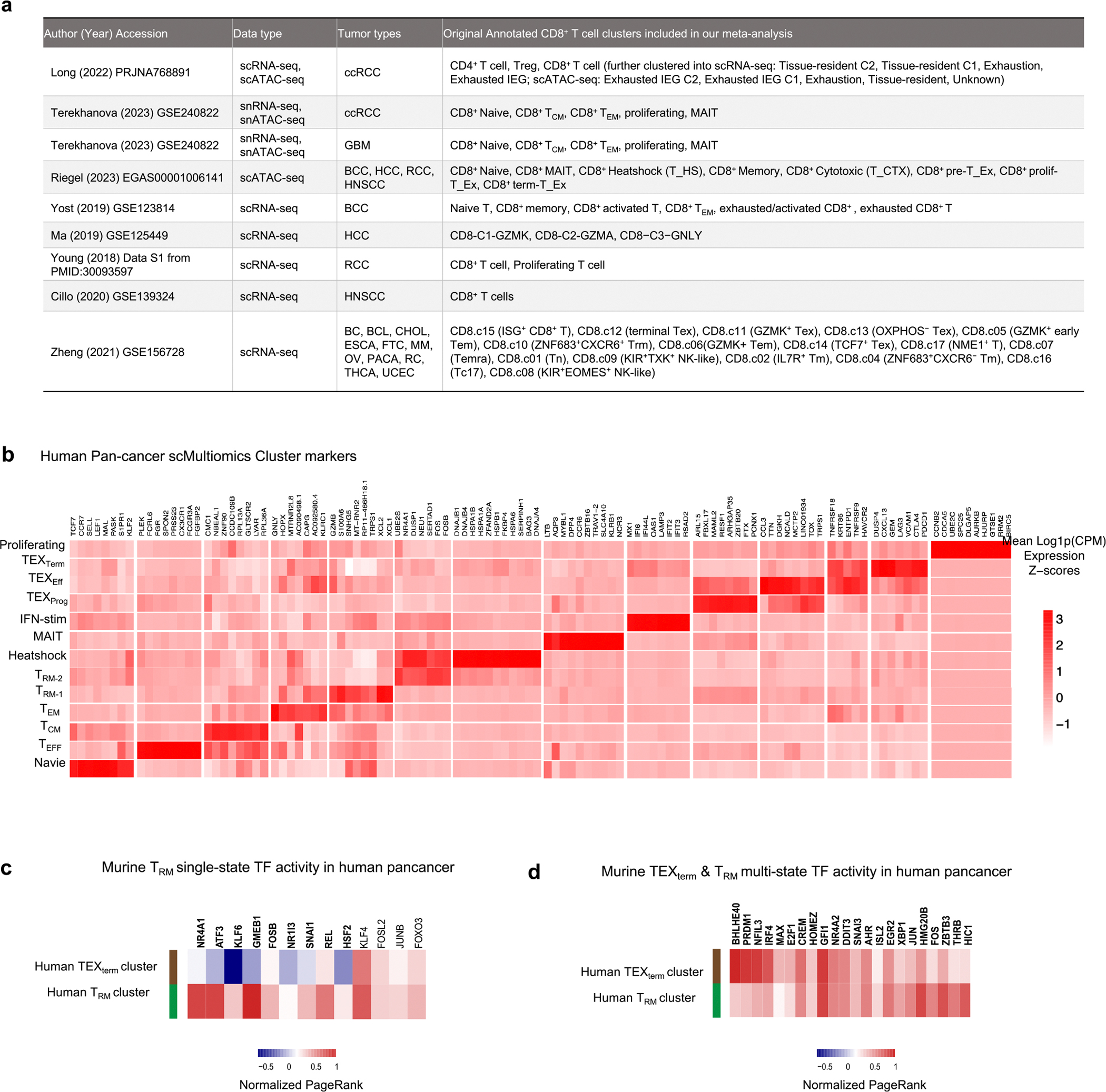
Conservation of single- and multi-state TFs in human pan-cancer TEX_term_ and T_RM_ cell states. **a**, Human pan-cancer datasets utilized in this study^48–55^. **b**, Cluster-specific marker mRNA expression across integrated pan-cancer single-cell multi-omics datasets. **c**, TF activity analysis using Taiji on matched matched scRNA-seq and scATAC-seq datasets from various human cancers, including ccRCC, GBM, BCC, HNSCC, HCC, and RCC. CD8^+^ T cell states were annotated using canonical marker gene expression. PageRank scores derived from Taiji were log-transformed, averaged per state, and z-score normalized. Results were visualized with a focus on TF activity T_RM_ and TEX_term_ cell states. **d**, mRNA expression of TFs were compared across human CD8^+^ T cell states. The dataset encompassed T cells from 15 tumor types. Cell type annotations provided by the original study were retained and mapped to the following broad categories of clusters: T_RM_, TEX_term_, T_EM_, Memory, Naive, and cell cycle. Seurat’s AverageExpression function was used, followed by z-score normalization. Data visualization emphasized comparisons between T_RM_ and TEX_term_ cell states. Cell-state-selectivity conserved TFs in humans and mice are highlighted in bold (**c, d**).

**Extended Data Fig. 10 |:**
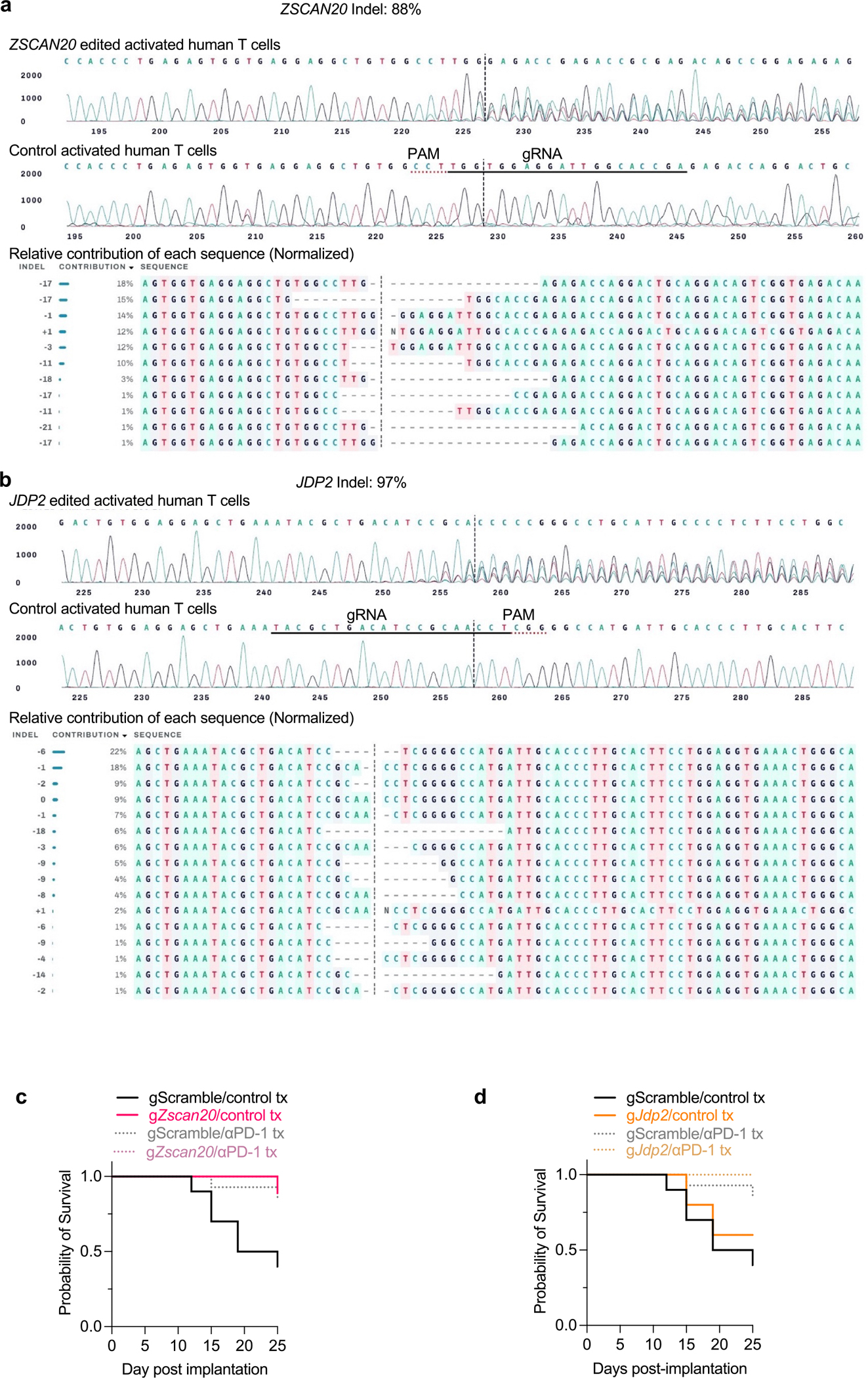
CRISPR validation of TEX_term_ single-state TF KO in human PBMCs and antitumor activity of TF-deficient T cells in mice. Indel frequencies, representative Sanger sequencing traces, and indel distributions for (**a**) *ZSCAN20* and (**b**) *JDP2* KOs. **c**, Survival of B16-GP33 bearing mice receiving *Zscan20* KO or control P14 CD8^+^ T cells, followed by anti-PD1 or isotype IgG2a treatment. **d**, Survival of tumor-bearing mice with *Jdp2* KO P14 CD8^+^ T cell transfer.

## Supplementary Material

Supplementary Methods

Supplementary Table 1

Reporting Summary

Supplementary Table 3

Supplementary Table 4

Supplementary Table 2

Supplementary Table 5

Supplementary Table 6

Supplementary Table 7

Supplementary Table 8

**Supplementary information** The online version contains supplementary material available at https://doi.org/10.1038/s41586-025-09989-7.

## Data Availability

ATAC-seq data from this paper will be deposited in the GEO database (GSE279498). Taiji v.2.0 output of this study (TF activity atlas, TF–TF interaction maps and TF activity on genome browser view) will be available at our CD8^+^ T cell TF atlas portal (https://wangweilab.shinyapps.io/Tcellstates/) and interactive interface for TF atlas exploration (https://huggingface.co/spaces/taijichat/chat). All other raw data are available from the corresponding author upon request. Source data are provided with this paper.

## Code availability

All scripts and the Taiji v.2.0 package are available at GitHub (https://github.com/Wang-lab-UCSD/Taiji2).
