# Publisher Correction: Atlas-guided discovery of transcription factors for T cell programming

**DOI:** 10.1038/s41586-026-10382-1

**Published:** 2026-03-18

**Authors:** H. Kay Chung, Cong Liu, Anamika Battu, Alexander N. Jambor, Brandon M. Pratt, Fucong Xie, Brian P. Riesenberg, Eduardo Casillas, Ming Sun, Elisa Landoni, Yanpei Li, Qidang Ye, Daniel Joo, Jarred Green, Zaid Syed, Nolan J. Brown, Matthew Smith, Shixin Ma, Shirong Tan, Brent Chick, Victoria Tripple, Z. Audrey Wang, Jun Wang, Bryan Mcdonald, Peixiang He, Qiyuan Yang, Timothy Chen, Siva Karthik Varanasi, Michael A. LaPorta, Thomas H. Mann, Dan Chen, Filipe Hoffmann, Josephine Ho, Jennifer Modliszewski, April Williams, Yusha Liu, Zhen Wang, Jieyuan Liu, Yiming Gao, Zhiting Hu, Ukrae H. Cho, Longwei Liu, Yingxiao Wang, Diana C. Hargreaves, Gianpietro Dotti, Barbara Savoldo, Jessica E. Thaxton, J. Justin Milner, Susan M. Kaech, Wei Wang

**Affiliations:** 1 NOMIS Center for Immunobiology and Microbial Pathogenesis, Salk Institute for Biological Studies, La Jolla, CA USA; 2 Lineberger Comprehensive Cancer Center, University of North Carolina at Chapel Hill, Chapel Hill, NC USA; 3 Department of Cell Biology and Physiology, University of North Carolina at Chapel Hill, Chapel Hill, NC USA; 4 Department of Chemistry and Biochemistry, University of California San Diego, La Jolla, CA USA; 5 Bioinformatics and Systems Biology Program, University of California San Diego, La Jolla, CA USA; 6 Department of Pharmacology, University of North Carolina at Chapel Hill, Chapel Hill, NC USA; 7 Molecular and Cell Biology Laboratory, Salk Institute for Biological Studies, La Jolla, CA USA; 8 Alfred E. Mann Department of Biomedical Engineering, University of Southern California, Los Angeles, CA USA; 9 Department of Bioengineering, Institute of Engineering in Medicine, University of California, La Jolla, CA USA; 10 Razavi Newman Integrative Genomics and Bioinformatics Core, Salk Institute for Biological Studies, La Jolla, CA USA; 11 Halıcıoğlu Data Science Institute, University of California San Diego, La Jolla, CA USA; 12 Department of Electrical and Computer Engineering, Texas A&M University, College Station, TX USA; 13 Department of Microbiology and Immunology, University of North Carolina at Chapel Hill, Chapel Hill, NC USA; 14 Department of Cellular and Molecular Medicine, University of California San Diego, La Jolla, CA USA; 15 Present Address: Institute for Immunology, Allen Institute, Seattle, WA USA

**Keywords:** Cellular immunity, Gene regulatory networks, Genomic engineering

Correction to: *Nature* 10.1038/s41586-025-09989-7 Published online 4 February 2026

In the version of this article initially published, Michael A. LaPorta’s name was listed incorrectly (Michael LaPorte) and is now amended in the HTML and PDF versions of the article.

