# Supplementary material for: Atlas-guided discovery of transcription factors for T cell programming
Source: Nature. Author manuscript; Available in PMC 2026 May 8. (PMC13017511; doi:10.1038/s41586-025-09989-7)
Supplement: Supplementary Methods [file NIHMS2159509-supplement-Supplementary_Methods.pdf]

nature portfolio

https://doi.org/10.1038/s41586-025-09989-7

Supplementary information

Atlas-guided discovery of transcription factors for T cell programming

In the format provided by the authors and unedited

Supplementary Method

TaijiChat: An Integrated Conversational Interface for Multi-Omics Data Exploration

To facilitate intuitive and interactive exploration of our Taiji multi-omics atlas, we developed TaijiChat, a web-based chatbot platform enabling natural language querying of both the study's datasets and associated literature. Unlike generic large language models (LLMs), TaijiChat provides domain-specific responses grounded in the content of our manuscript, supplementary materials, and underlying transcriptomic and epigenomic datasets. This tight integration with structured outputs—such as TF activity scores, cell clustering results, and motif enrichment profiles—enables users to dynamically engage in context-aware dialogues with the data. Users can directly query TF activity (e.g., "Which TFs are enriched in TRM cells in GBM?"), request visualizations (e.g., UMAP plots or violin plots of selected genes), or retrieve ranked TF lists by specific cell states or tumor contexts.

In addition to data interrogation, TaijiChat incorporates a literature-aware component. Each generated response is cross-referenced with relevant supporting or contrasting studies from databases such as PubMed and GEO, allowing users to validate findings, assess consistency with prior work, and develop novel hypotheses. For example, querying the role of KLF6 in TRM differentiation yields not only a summary of its activity across tumor-infiltrating lymphocytes (TILs) and associated figures from our atlas, but also citations to independent literature corroborating its specific function in TRM cells.

To systematically enable this advanced functionality, we designed TaijiChat as a sophisticated multi-agent artificial intelligence system, integrating natural language processing, safety-verified code generation, and domain-specific expertise.

The system employs a manager-orchestrated architecture consisting of specialized agents responsible for planning, validation, and secure code execution. Specifically, this architecture embodies four core design principles critical for scientific computing applications:

1. Safety-first architecture: Ensures all generated analytical code undergoes mandatory validation through multiple security layers prior to execution.
2. Separation of concerns: Assigns distinct, specialized roles to individual agents, facilitating modularity, scalability, and maintainability.
3. Intelligent orchestration: Provides centralized coordination of complex multi-step workflows, ensuring coherent interactions across system components.
4. Domain expertise integration: Directly incorporates methodologies and domain-specific knowledge into the reasoning and analysis processes, enabling contextually appropriate responses.

User guide

1) Access TaijiChat: After logging in, navigate to the TaijiChat testing page (https://huggingface.co/spaces/taijichat/test-taijichat)
2) Initial Loading: Upon your first visit, TaijiChat will automatically load necessary packages and build the system from scratch. This initial setup may take a few minutes. You will know TaijiChat is ready when a green "running" indicator appears in the top-left corner of the webpage.
3) Testing Chat Functionality: Once TaijiChat is running, select the "Chat" tab on the website. You may then begin entering questions to test the system. Note that the first query might respond slowly due to system initialization.

4) Accuracy and Limitations: TaijiChat is powered by state-of-the-art Large Language Models (LLMs), which can still occasionally make mistakes. Always verify critical scientific information against original research papers and authoritative sources.

System evaluation

To evaluate TaijiChat's effectiveness, we curated a benchmark set of 21 expert-driven immunology questions, comparing responses from TaijiChat to those from a general-purpose LLM lacking direct data integration. TaijiChat demonstrated accurate, evidence-supported responses in all tested scenarios, highlighting its utility as a domain-specific scientific assistant.

Collectively, TaijiChat represents a platform for hypothesis generation and data interpretation, effectively combining the intuitive accessibility of conversational AI with rigorous, integrated multi-omics data analysis and automated literature synthesis.

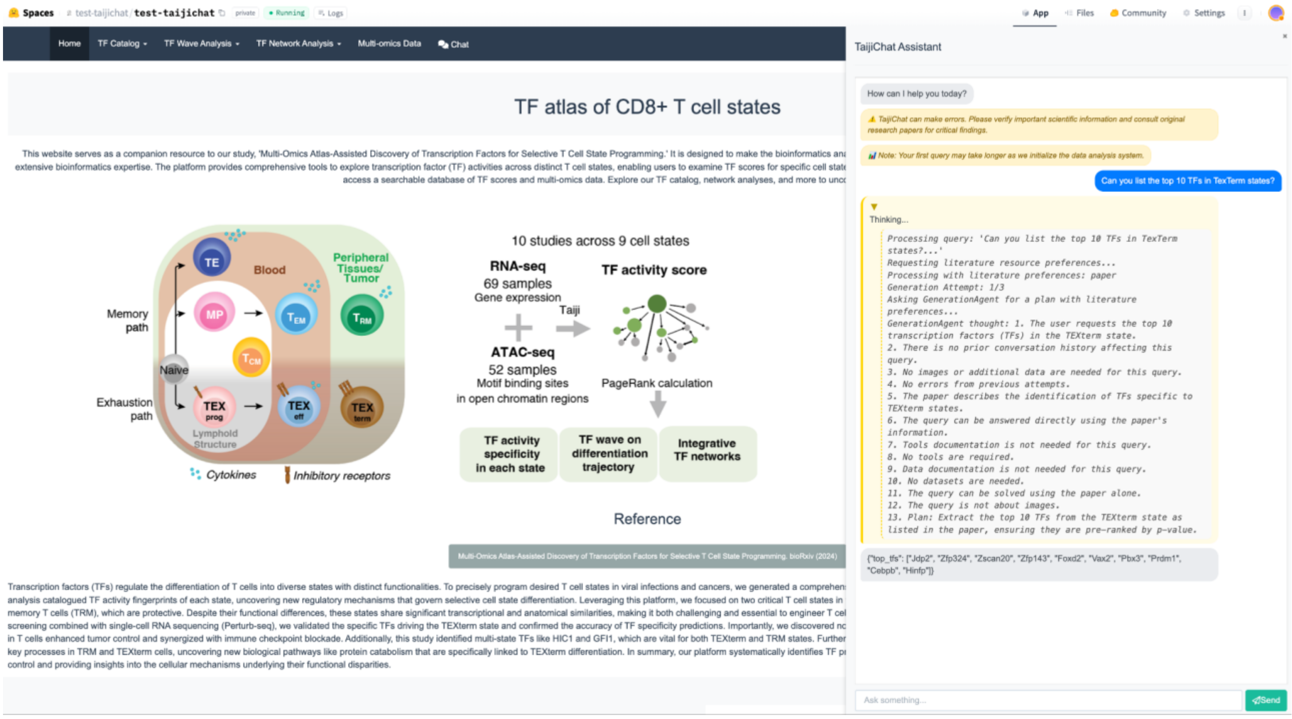

<u>*Agent Architecture and Coordination:*</u> The system consists of four specialized agents operating under centralized coordination: the Manager Agent, Generation Agent, Supervisor Agent, and Executor Agent.

The Manager Agent serves as the central orchestrator in a multi-agent architecture designed for scientific computing and code generation tasks. The system employs a hierarchical workflow where the Manager coordinates three specialized sub-agents: a GenerationAgent that transforms natural language queries into executable Python code and structured execution plans, a SupervisorAgent that performs safety validation and code review to prevent potentially harmful operations, and an ExecutorAgent that executes approved code in a controlled environment. The Manager implements a sophisticated query processing pipeline with built-in retry logic (up to three attempts) to handle generation failures, maintains conversation history for context-aware responses, and supports multi-step computational workflows where intermediate results inform subsequent processing steps. This design ensures robust, safe, and efficient processing of complex scientific queries while maintaining clear separation of concerns across the agent hierarchy.RetryClaude can make mistakes.

The Generation Agent functions as the intelligent planner, employing a structured 13-step reasoning process for query understanding and solution development. This agent integrates domain-specific knowledge of transcription factor biology and experimental methodologies, enabling scientifically appropriate interpretations of natural language queries. The reasoning framework encompasses query decomposition, conversation history integration, multi-modal content evaluation, scientific domain knowledge activation, tool capability assessment, and comprehensive execution plan synthesis.

The Supervisor Agent implements a critical safety validation layer through multi-stage code review processes. This agent performs static code analysis, security pattern detection, and tool usage verification before any code execution. The validation process examines code structure, prevents dangerous system operations, validates tool usage patterns, and provides detailed feedback for rejected code with specific remediation suggestions.

The Executor Agent provides secure code execution within a sandboxed Python environment. This agent implements restricted global scope access, limiting available modules to approved scientific computing libraries and custom tools. All code execution occurs in isolation with comprehensive output capture and sanitized error reporting, preventing system exposure while maintaining informative debugging capabilities.

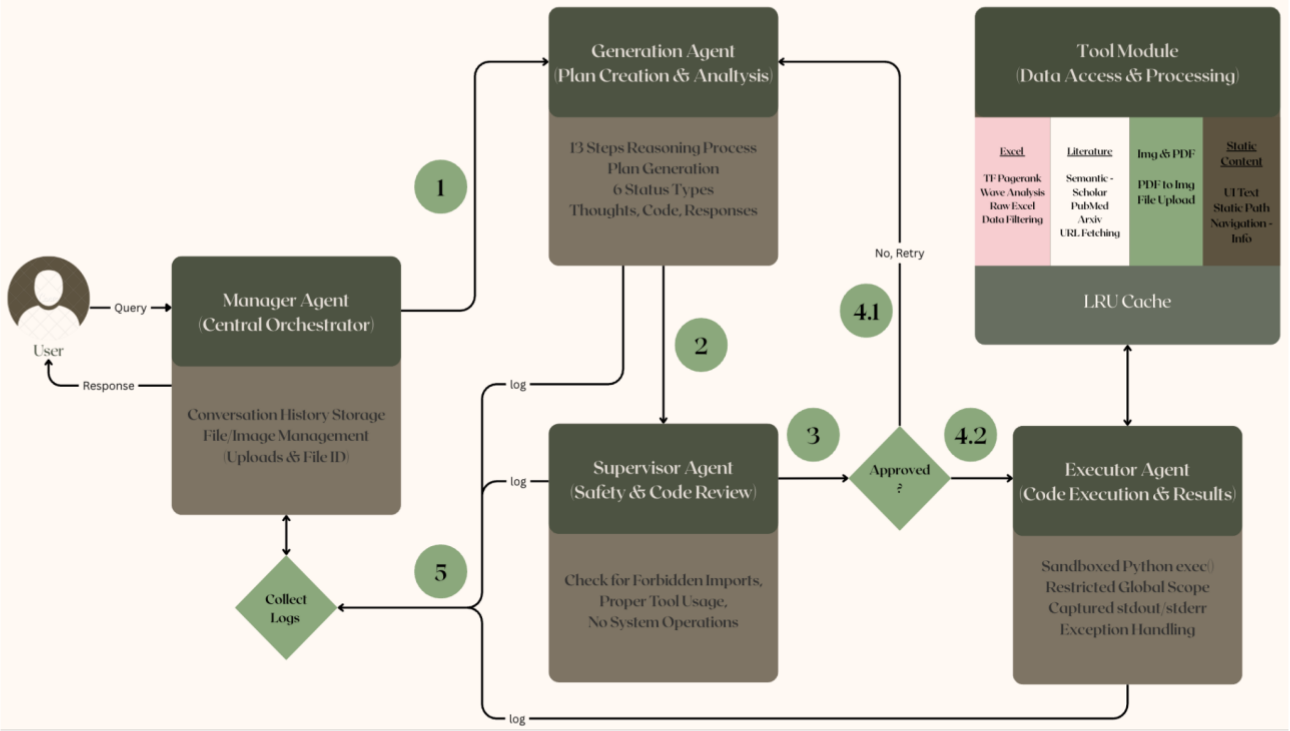

<u>Safety and Security Architecture:</u> The system implements a three-layer security model designed to prevent unsafe operations while maintaining scientific functionality. Layer 1 provides generation-time safety through security-aware code templates and built-in constraint knowledge within the Generation Agent. Layer 2 implements mandatory pre-execution review through the Supervisor Agent's pattern-based security analysis and tool compliance verification. Layer 3 enforces execution isolation through the Executor Agent's sandboxed environment with restricted resource access and output sanitization. Error recovery mechanisms include intelligent retry strategies with supervisor feedback integration, progressive query simplification for complex

requests, and maintenance of conversation context across retry attempts. The system limits retry attempts to three per query while incorporating learned patterns from previous failures to improve subsequent attempts.

Tools Integration and Performance Optimization: The system interfaces with a comprehensive tools module providing specialized functions for scientific data analysis. Data processing capabilities include Excel file processing with proper formatting, temporal pattern analysis, access to correlation matrices, and intelligent data subsetting. Literature integration tools provide multi-source academic database searches across PubMed, ArXiv, and Semantic Scholar, with automated content extraction and deduplication algorithms. Multi-modal analysis capabilities support AI-powered image analysis, PDF document processing, and file format detection. Performance optimization employs a multi-level caching strategy including function-level LRU caching, data-level timeout caching, schema caching for persistent structures, and session-based query-response caching. Resource efficiency measures include lazy loading of datasets, batch processing for large operations, automatic memory management, and intelligent API rate limiting for external services

Technical Implementation and Performance: The system utilizes OpenAI GPT-4o models for both text and vision processing, operating within a Python 3.x runtime environment with scientific computing libraries including pandas and openpyxl. External API integration provides access to major academic databases while maintaining security through input validation and sandboxed execution.

Architectural Advantages for Scientific Applications: This multi-agent architecture provides several key advantages for scientific computing applications. The multi-layer validation system ensures research integrity through multiple checkpoints, preventing unsafe operations while maintaining detailed audit trails of all agent interactions. The modular design enables easy maintenance and extensibility, allowing individual agent updates without system-wide modifications and seamless integration of new analytical capabilities. Scientific rigor is emphasized through reproducible operations with consistent outputs, transparent reasoning traces for scientific validation, comprehensive academic literature integration, and built-in domain expertise for methodologically appropriate analyses. The system's fail-safe design defaults to safe states during error conditions while providing robust error recovery with intelligent retry mechanisms.
