# Supplementary material for: Atlas-guided discovery of transcription factors for T cell programming
Source: Nature. Author manuscript; Available in PMC 2026 May 8. (PMC13017511; doi:10.1038/s41586-025-09989-7)
Supplement: Reporting Summary [file NIHMS2159509-supplement-Reporting_Summary.pdf]

nature portfolio

Corresponding author(s): Susan Kaech, Wei Wang, H.Kay Chung

Last updated by author(s): 10/24/2025

Reporting Summary

Nature Portfolio wishes to improve the reproducibility of the work that we publish. This form provides structure for consistency and transparency in reporting. For further information on Nature Portfolio policies, see our Editorial Policies and the Editorial Policy Checklist.

Statistics

For all statistical analyses, confirm that the following items are present in the figure legend, table legend, main text, or Methods section.

n/a	Confirmed	
☐	☒	The exact sample size (*n*) for each experimental group/condition, given as a discrete number and unit of measurement
☐	☒	A statement on whether measurements were taken from distinct samples or whether the same sample was measured repeatedly
☐	☒	The statistical test(s) used AND whether they are one- or two-sided<br>*Only common tests should be described solely by name; describe more complex techniques in the Methods section.*
☐	☒	A description of all covariates tested
☐	☒	A description of any assumptions or corrections, such as tests of normality and adjustment for multiple comparisons
☐	☒	A full description of the statistical parameters including central tendency (e.g. means) or other basic estimates (e.g. regression coefficient) AND variation (e.g. standard deviation) or associated estimates of uncertainty (e.g. confidence intervals)
☐	☒	For null hypothesis testing, the test statistic (e.g. *F*, *t*, *r*) with confidence intervals, effect sizes, degrees of freedom and *P* value noted<br>*Give P values as exact values whenever suitable.*
☒	☐	For Bayesian analysis, information on the choice of priors and Markov chain Monte Carlo settings
☐	☒	For hierarchical and complex designs, identification of the appropriate level for tests and full reporting of outcomes
☐	☒	Estimates of effect sizes (e.g. Cohen's *d*, Pearson's *r*), indicating how they were calculated

Our web collection on statistics for biologists contains articles on many of the points above.

Software and code

Policy information about availability of computer code

Data collection	Data acquisition was performed on a LSR II (BD), Cytek Aurora, Cytek Northern Lights, and analysis was performed using Flowjo software (TreeStar). All sorting was performed on BD Aria or BD Influx.
Data analysis	FlowJo v10 (FlowJo LLC, USA) was used for analysis of cytometric data. Statistical analysis was performed using GraphPad version 9 (Prism Software Inc., USA) and R version 4.2.0 (https://www.r-project.org).Taiji v2(https://github.com/Wang-lab-UCSD/Taiji2) and Seurat v4.1.1.

For manuscripts utilizing custom algorithms or software that are central to the research but not yet described in published literature, software must be made available to editors and reviewers. We strongly encourage code deposition in a community repository (e.g. GitHub). See the Nature Portfolio guidelines for submitting code & software for further information.

Data

Policy information about availability of data

All manuscripts must include a data availability statement. This statement should provide the following information, where applicable:

- Accession codes, unique identifiers, or web links for publicly available datasets
- A description of any restrictions on data availability
- For clinical datasets or third party data, please ensure that the statement adheres to our policy

sequencing data were deposited GSE279498 (reviewer token: idgfiqgozvsnnuh)

Research involving human participants, their data, or biological material

Policy information about studies with human participants or human data. See also policy information about sex, gender (identity/presentation), and sexual orientation and race, ethnicity and racism.

Reporting on sex and gender	*Use the terms sex (biological attribute) and gender (shaped by social and cultural circumstances) carefully in order to avoid confusing both terms. Indicate if findings apply to only one sex or gender; describe whether sex and gender were considered in study design; whether sex and/or gender was determined based on self-reporting or assigned and methods used. Provide in the source data disaggregated sex and gender data, where this information has been collected, and if consent has been obtained for sharing of individual-level data; provide overall numbers in this Reporting Summary. Please state if this information has not been collected. Report sex- and gender-based analyses where performed, justify reasons for lack of sex- and gender-based analysis.*
Reporting on race, ethnicity, or other socially relevant groupings	*Please specify the socially constructed or socially relevant categorization variable(s) used in your manuscript and explain why they were used. Please note that such variables should not be used as proxies for other socially constructed/relevant variables (for example, race or ethnicity should not be used as a proxy for socioeconomic status). Provide clear definitions of the relevant terms used, how they were provided (by the participants/respondents, the researchers, or third parties), and the method(s) used to classify people into the different categories (e.g. self-report, census or administrative data, social media data, etc.) Please provide details about how you controlled for confounding variables in your analyses.*
Population characteristics	*Describe the covariate-relevant population characteristics of the human research participants (e.g. age, genotypic information, past and current diagnosis and treatment categories). If you filled out the behavioural & social sciences study design questions and have nothing to add here, write "See above."*
Recruitment	*Describe how participants were recruited. Outline any potential self-selection bias or other biases that may be present and how these are likely to impact results.*
Ethics oversight	*Identify the organization(s) that approved the study protocol.*

Note that full information on the approval of the study protocol must also be provided in the manuscript.

Field-specific reporting

Please select the one below that is the best fit for your research. If you are not sure, read the appropriate sections before making your selection.

☒ Life sciences ☐ Behavioural & social sciences ☐ Ecological, evolutionary & environmental sciences

For a reference copy of the document with all sections, see nature.com/documents/nr-reporting-summary-flat.pdf

Life sciences study design

All studies must disclose on these points even when the disclosure is negative.

Sample size	Sample size is indicated in the figure legends and was determined based on prior studies from our lab. No statistical methods were used to predetermine sample sizes but our samples sizes are similar to those reported in prior publications.
Data exclusions	No data were excluded.
Replication	All mouse experiments were successfully repeated ≥2 times and where possible quantification and statistics were run on combined replicate experiments.
Randomization	In all experiments age and sex-matched mice were randomly assigned to experimental groups. All experiments involved control samples and the respective treatment conditions.
Blinding	No blinding was performed.

Reporting for specific materials, systems and methods

We require information from authors about some types of materials, experimental systems and methods used in many studies. Here, indicate whether each material, system or method listed is relevant to your study. If you are not sure if a list item applies to your research, read the appropriate section before selecting a response.

Materials & experimental systems

n/a	Involved in the study	
☐	☒	Antibodies
☐	☒	Eukaryotic cell lines
☒	☐	Palaeontology and archaeology
☐	☒	Animals and other organisms
☒	☐	Clinical data
☒	☐	Dual use research of concern
☒	☐	Plants

Methods

n/a	Involved in the study	
☒	☐	ChIP-seq
☐	☒	Flow cytometry
☒	☐	MRI-based neuroimaging

Antibodies

Antibodies used

TCF1, PacificBlue, C63D9, Cell Signaling, 9066S;
CX3CR1, PerCP/Cyanine5.5, SA011F11, Biolegend, 149010;
CX3CR1, APC/Fire™ 750, SA011F11, Biolegend, 149040;
Tim3, APC, RMT3-23, Biolegend, 119721;
Tim3, BV421, RMT3-23, Biolegend, 119723;
PD-1, BV785, 29F.1A12, Biolegend, 135225;
Thy1.1, A488, OX-7, Biolegend, 202506;
Thy1.2, PerCP-Cy5.5, 30-H12, Biolegend, 105322;
Ly5.1, BUV737, A20, Biolegend, 110708;
Ly5.2, BUV395, A20, Biolegend, 110708;
CD101, PE, Moushi101, Invitrogen, 12-1011-82;
CD101, PeCy7, Moushi101, eBioscience, 25-1011-80;
SLAMF6, BV605, 13G3, BD, 745250;
CD39, Pe-Cy7, 24DMS1, eBioscience, 25-0391-82;
CD38, PerCP-Cy5.5, 24DMS1, eBioscience, 25-0391-82;
CXCR6, PE, SA051D1, Biolegend, 151104;
KLRG1, PeCy7, 2F1, Biolegend, 138416;
KLRG1, FITC,2F1/KLRG1, Biolegend, 138410;
KLRG1, APC/Cy7,2F1/KLRG1, Biolegend, 138426;
CD127, BV421, A7R34, Biolegend, 135024;
CD8a, BUV395, 53-6.7, BD, 565968;
CD8a, BV711, 53-6.7, Biolegend, 100747;
CD69, PE-Cy7, H1.2F3, Biolegend 104512;
CD103, APC, M290, BD, 562772;
IFNg, PE-Cy7, XMG1.2, Biolegend, 505826;
TNF, BV421, MP6-XT22, Biolegend, 506328;
TOX, e660, TXRX10, Thermo Fisher Scientific, 50-6502-82;
TruStain FcX™ (anti-mouse CD16/32), Biolegend, 101320;
CD8, BUV395, RPA-T8, BD, 563796;
CD8, AF700, RPA-T8, Biolegend, 301028;
CD8, BUV496, RPA-T8, BD, 612942;
CD4, BUV737, SK3, BD, 612748;
CD4, BV711, SK3,;
CD45RA, FITC, H100, Biolegend, 304106;
CD45RA, BV785, H100, Biolegend, 304140;
CD45RO, PE, UCHL1, Biolegend, 304206;
CD45RO, BV711, UCHL1, Biolegend, 304236;
CD45RO, BV605, UCHL1, Biolegend, 304238;
CCR7, APC/Cy7, G043H7, Biolegend, 353212;
CD62L, PE, DREG-56, Biolegend, 304805;
CD69, FITC, FN50, Biolegend, 310904;
CD69, APC/Cy7, FN50, Biolegend, 310913;
CD103, APC, Ber-ACT8, Biolegend, 350216;
CD103, BV421, Ber-ACT8,,;
CXCR6, PCP/Cy5.5, K041E5, Biolegend, 356010;
PD1, BV421, EH12.2H7, BD, 562516;
PD1, FITC, EH12.2H7, Biolegend, 329904;
PD1, BUV737, EH12.2H7, BD, 612791;
CD39, BV605, A1, BD, 567691;
CD39, PCP/Cy5.5, A1, Biolegend, 328218;
LAG3, PE/Cy7, 11C3C65, Biolegend, 369310;
LAG3, Spark Plus UV395, 11C3C65, Biolegend, 369354;
TIM3, BV711, F38-2E2, Biolegend, 345024;
TIGIT, APC, A15153G, Biolegend, 372706;
TIGIT, PE, A15153G, Biolegend, 372704;
IFN-γ, PE, 4S.B3, Biolegend, 502509;
IFN-γ, BV605, 4S.B3, Biolegend, 506542;
TNF-α, BV785, MAb11, Biolegend, 502948;

TNF-α, APC, MAb11, Biolegend, 502913;
IL 2, FITC, MQ1-17H12, Biolegend, 500304;
IL 2, RB780, MQ1-17H12, Biolegend, 569130;
GZMB, APC, QA16A02, Biolegend, 372204;
GZMB, AF700, QA16A02, Biolegend, 372222;
G4S Linker, PE, E7O2V, Cell Signaling, 38907S;
G4S Linker, AF594, E7O2V, Cell Signaling, 39614S;
G4S Linker, Pacific Blue, E7O2V, Cell Signaling, 44962S;
CD56, BUV563, NCAM16.2, BD, 612929;

Validation

All antibodies were acquired from commercial sources and have been validated by the vendors. Validation data are available on the manufacturer's website.

Eukaryotic cell lines

Policy information about cell lines and Sex and Gender in Research

Cell line source(s)

HEK293T cells, B16-GP33 cells

Authentication

None of the cell lines were authenticated.

Mycoplasma contamination

All cell lines tested negative for mycoplasma by PCR prior to use.

Commonly misidentified lines
(See ICLAC register)

No commonly misidentified lines were used in this study.

Animals and other research organisms

Policy information about studies involving animals; ARRIVE guidelines recommended for reporting animal research, and Sex and Gender in Research

Laboratory animals

C57BL/6J mice were purchased from Jackson Laboratories and UNC Animal Models Core. P14+ mice have been previously described (Pircher, Nature 1989). P14+ Thy1.1, P14+ Ly5.1, P14+ Cas9 Ly5.1 were breed in house. Animals were housed in specific-pathogen-free facilities at the Salk Institute and UNC Chapel Hill. All experimental studies were approved and performed in accordance with guidelines and regulations implemented by the Salk Institute Animal Care and Use Committee and University of North Carolina at Chapel Hill Animal Care and Use Committee.

Wild animals

This study did not involve wild animals.

Reporting on sex

Both male and female mice between 6-12 weeks of age were used.

Field-collected samples

This study did not involve field-collected samples.

Ethics oversight

All experimental studies were approved and performed in accordance with guidelines and regulations implemented by the Salk Institute and UNC Chapel Hill Animal Care and Use Committee.

Note that full information on the approval of the study protocol must also be provided in the manuscript.

Plants

Seed stocks

Report on the source of all seed stocks or other plant material used. If applicable, state the seed stock centre and catalogue number. If plant specimens were collected from the field, describe the collection location, date and sampling procedures.

Novel plant genotypes

Describe the methods by which all novel plant genotypes were produced. This includes those generated by transgenic approaches, gene editing, chemical/radiation-based mutagenesis and hybridization. For transgenic lines, describe the transformation method, the number of independent lines analyzed and the generation upon which experiments were performed. For gene-edited lines, describe the editor used, the endogenous sequence targeted for editing, the targeting guide RNA sequence (if applicable) and how the editor was applied.

Authentication

Describe any authentication procedures for each seed stock used or novel genotype generated. Describe any experiments used to assess the effect of a mutation and, where applicable, how potential secondary effects (e.g. second site T-DNA insertions, mosiacism, off-target gene editing) were examined.

Flow Cytometry

Plots

Confirm that:

☒ The axis labels state the marker and fluorochrome used (e.g. CD4-FITC).

☒ The axis scales are clearly visible. Include numbers along axes only for bottom left plot of group (a 'group' is an analysis of identical markers).

☒ All plots are contour plots with outliers or pseudocolor plots.

☒ A numerical value for number of cells or percentage (with statistics) is provided.

Methodology

Sample preparation	Single-cell suspensions of splenocytes were prepared by mechanically disaggregating spleens through a 70 µm cell strainer (VWR), followed by red blood cell lysis using ACK lysis buffer (KD Medical). Intraepithelial lymphocyte (IEL) cells were isolated from the small intestine, after removing Peyer's patches, by incubating the tissue in HEPES buffer containing FBS and DTT solution. IELs were then purified using a Percoll gradient, 21 days post-LCMV Armstrong infection.<br><br>Tumor-infiltrating T cells were harvested 21 days after tumor implantation. Tumor tissue was minced using razor blades in a cell culture dish and digested with a dissociation buffer. The dissociation buffer (10x) was composed of 40 ml RPMI/DMEM (Gibco), 1% Pen/Strep (Gibco), 1 mM sodium pyruvate (Gibco), 25 mM HEPES (Lonza), 400 mg Collagenase IV (Sigma), 400 mg Soybean Trypsin Inhibitor (Thermo Scientific), 50 mg Dispase II (Sigma), and 20 mg DNase (Sigma). The digestion was performed for 30 minutes at 37°C. After digestion, the samples were passed through a 70 µm cell strainer, centrifuged at 420 rcf for 4 minutes at 4°C, and resuspended in RPMI supplemented with 10% fetal bovine serum prior to staining.
Instrument	Data acquisition was performed on a LSR II (BD), Cytek Aurora, Cytek Northern light, and analysis was performed using Flowjo software (TreeStar). All sorting was performed on BD Aria or BD Influx.
Software	FlowJo v10 (FlowJo LLC, USA) was used for analysis of cytometric data.
Cell population abundance	The purity of sorted samples was typically >95%. To check purity, an aliquot of sorted cells was analyzed.
Gating strategy	Cells were gated in FSCxSSC for lymphocytes, followed by two singlet discrimination gates and exclusion of dead cells using live/dead dye. P14+ cells were identified based on congenics, endogenous antigen-specific cells were identified based on tetramer positivity. Transduced cells were gated on GFP.

☐ Tick this box to confirm that a figure exemplifying the gating strategy is provided in the Supplementary Information.
